# CCL3 in the bone marrow microenvironment causes bone loss and bone marrow adiposity in aged mice

**DOI:** 10.1172/jci.insight.159107

**Published:** 2023-01-10

**Authors:** Degang Yu, Shuhong Zhang, Chao Ma, Sen Huang, Long Xu, Jun Liang, Huiwu Li, Qiming Fan, Guangwang Liu, Zanjing Zhai

**Affiliations:** 1 Shanghai Key Laboratory of Orthopedic Implants, Department of Orthopedic Surgery, Shanghai Ninth People’s Hospital, Shanghai Jiao Tong University School of Medicine, Shanghai, China.; 2 Department of Orthopedics, Xuzhou Central Hospital, Xuzhou Clinical School of Xuzhou Medical University; Xuzhou Central Hospital Affiliated to Nanjing University of Chinese Medicine, The Xuzhou School of Clinical Medicine of Nanjing Medical University; and Xuzhou Central Hospital Affiliated to Medical School of Southeast University, Xuzhou, China.

**Keywords:** Aging, Bone Biology, Bone marrow differentiation, Chemokines, Osteoporosis

## Abstract

The central physiological role of the bone marrow renders bone marrow stromal cells (BMSCs) particularly sensitive to aging. With bone aging, BMSCs acquire a differentiation potential bias in favor of adipogenesis over osteogenesis, and the underlying molecular mechanisms remain unclear. Herein, we investigated the factors underlying age-related changes in the bone marrow and their roles in BMSCs’ differentiation. Antibody array revealed that CC chemokine ligand 3 (CCL3) accumulation occurred in the serum of naturally aged mice along with bone aging phenotypes, including bone loss, bone marrow adiposity, and imbalanced BMSC differentiation. In vivo *Ccl3* deletion could rescue these phenotypes in aged mice. CCL3 improved the adipogenic differentiation potential of BMSCs, with a positive feedback loop between CCL3 and C/EBPα. CCL3 activated C/EBPα expression via STAT3, while C/EBPα activated CCL3 expression through direct promoter binding, facilitated by DNA hypomethylation. Moreover, CCL3 inhibited BMSCs’ osteogenic differentiation potential by blocking β-catenin activity mediated by ERK-activated Dickkopf-related protein 1 upregulation. Blocking CCL3 in vivo via neutralizing antibodies ameliorated trabecular bone loss and bone marrow adiposity in aged mice. This study provides insights regarding age-related bone loss and bone marrow adiposity pathogenesis and lays a foundation for the identification of new targets for senile osteoporosis treatment.

## Introduction

Human aging is associated with osteoporosis characterized by bone loss, bone fragility, and fractures. Hence, the cellular and molecular mechanisms of age-related bone loss are areas of intensive investigation. In postnatal organisms, bone formation is dependent on bone marrow stromal cells (BMSCs). Skeletal stem cells are a BMSC subset that are recruited to the bone formation surface during bone remodeling (1). BMSCs have the ability to self-renew and differentiate into multiple lineages, including osteoblasts, adipocytes, chondrocytes, and hematopoiesis-supportive stroma (2–4). BMSCs are sensitive to aging and age-related diseases due to their central role in coordinating bone marrow activity. Reciprocally, changes in BMSCs’ activities and functions could contribute to bone marrow aging.

Aging triggers a decrease in trabecular bone mass and an increase in bone marrow adipocytes, both of which are more pronounced in patients with osteoporosis (5, 6). However, during senile osteoporosis, the rate of adipogenesis increases, while that of osteogenesis decreases (7). Moreover, BMSCs isolated from aged mice exhibit high efficiency of adipocyte differentiation as compared with osteoblasts and vice versa (8). These findings have led to the hypothesis that a deficiency in the number and function of osteoblasts and an increase in marrow adipogenesis represent key etiological factors of senile osteoporosis. More specifically, the potential of BMSCs to differentiate in an unbalanced manner into adipocytes and osteoblasts can cause bone loss and excessive marrow adipocyte accumulation (9). However, the molecular mechanisms underlying these phenomena remain unknown.

The differentiation potential of BMSCs is determined by intrinsic and extrinsic factors. The former comprises all the structural and functional components of BMSCs, including genomic, transcriptomic, proteomic, and epigenomic factors. Meanwhile, extrinsic factors comprise the complex bone marrow microenvironment surrounding BMSCs, composed of stromal cells, extracellular matrix, and various soluble cytokines (9). BMSCs’ differentiation is initiated by regulatory signals from the bone marrow microenvironment. Indeed, age-related changes in the bone microenvironment may contribute to aging-related decreases in bone formation. However, it is unclear which cell types are responsible for changes in growth factor secretion. Hence, detection of bone marrow factors that undergo abnormal age-related changes and characterization of their roles in the modulation of BMSC osteogenic and adipogenic differentiation potential are vital to elucidating the molecular mechanisms underlying age-related bone diseases.

CC chemokine ligand 3 (CCL3) — originally named macrophage inflammatory protein 1α (10) — is a proinflammatory cytokine that induces the chemotaxis of monocytes and T and B cells, is involved in the inflammatory process (11), suppresses hematopoietic stem cell (HSC) proliferation (12), and is a potent osteoclast activator (13). Chemokines from one subfamily bind only to specific receptors from the corresponding family (14). CCL3, for example, selectively activates CCR1 and CCR5 (14). Prior studies have focused on the role of CCL3 in multiple myeloma (MM), reporting that CCL3 is produced by MM cells and directly promotes osteoclast formation and differentiation (15). Patients with postmenopausal osteoporosis (type I osteoporosis with high bone turnover) exhibit increased serum CCL3 levels compared with postmenopausal nonosteoporotic and control individuals (16). Indeed, serum CCL3 levels negatively correlate with bone mineral density at the total hip, femoral neck, and L1–L4 lumbar spine (16). CCL3 inhibits osteoblast proliferation and decreases their osteogenic differentiation potential by downregulating osteocalcin (*Ocn*), runt-related transcription factor 2 (*Runx2*), and osterix (*Osx*) expression (17). However, no studies have reported the role of CCL3 in senile osteoporosis, i.e., type II osteoporosis with low bone turnover.

In the current study, we revealed for the first time to our knowledge that CCL3 accumulates in the bone marrow of aged mice exhibiting bone loss and bone marrow adiposity. In vivo ablation of CCL3 abrogated the bone loss and bone marrow adiposity of aged mice. CCL3 promoted adipogenic differentiation by driving a positive feedback loop between CCL3 and C/EBPα and inhibiting the osteogenic differentiation of BMSCs by ERK-mediated Dickkopf-related protein 1 (DKK1) upregulation. Importantly, we found that in vivo blocking of CCL3 with neutralization antibodies partially ameliorated trabecular bone loss and bone marrow adiposity of aged mice. Our study provides novel insights regarding the pathogenesis of age-related bone loss and bone marrow adiposity, while laying the groundwork for future research to identify new targets to treat senile osteoporosis.

## Results

### Bone phenotypes, bone marrow adiposity, and imbalance of BMSCs’ in vitro osteogenic and adipogenic differentiation potential in aged mice.

The peak bone mass of mice is achieved between 5 and 6 months of age (18), followed by progressive decline with aging (19). Skeletal aging occurs in 18- to 22-month-old mice (20). Thus, 6- and 18-month-old mice were used as young and aged mice, respectively, in the current study.

**Figure 1:**
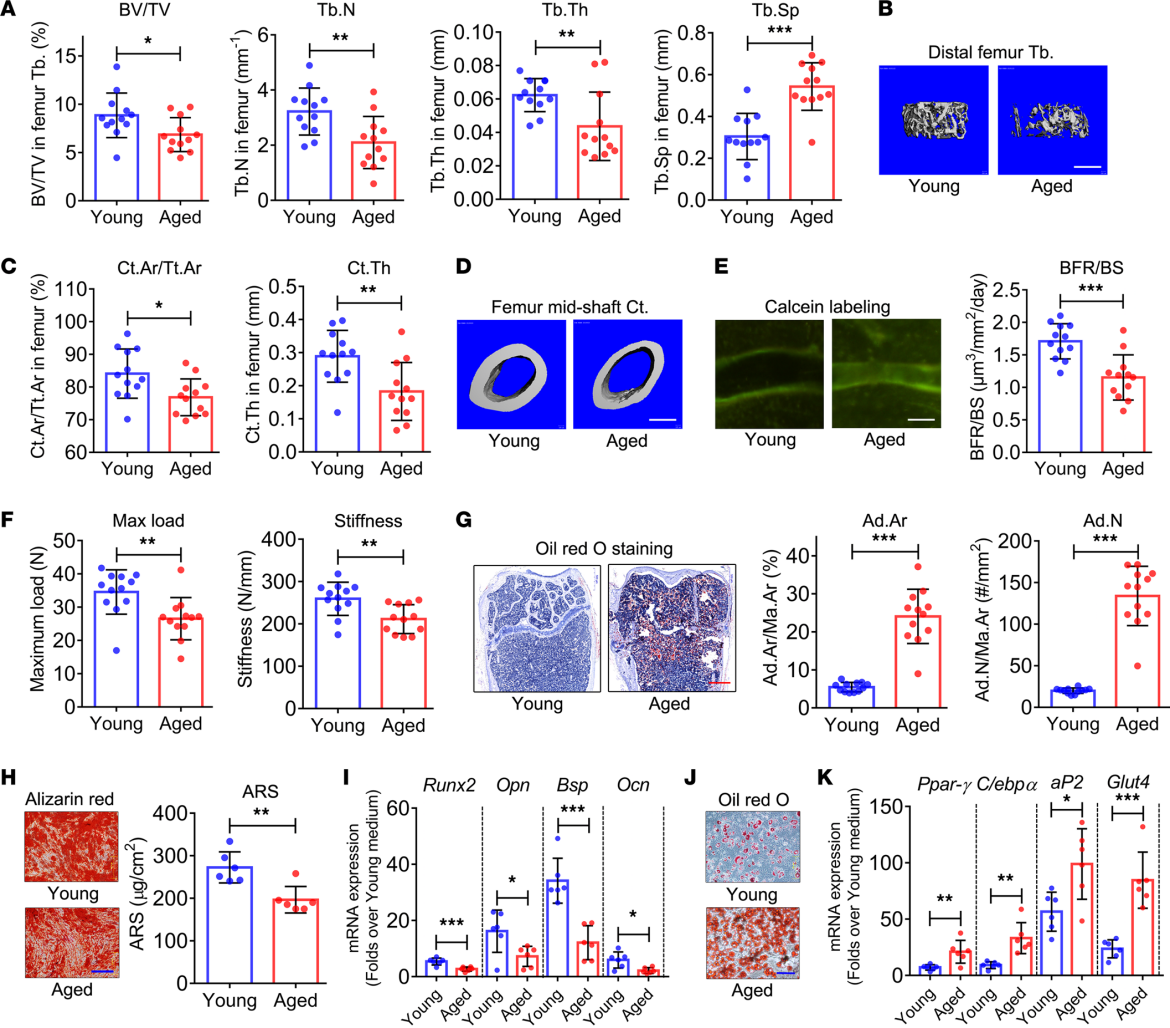
Bone phenotypes, bone marrow adiposity, and imbalance of in vitro osteogenic and adipogenic differentiation potential of BMSCs in aged mice. (**A**) Trabecular bone volume fraction (BV/TV), trabecular bone number (Tb.N), trabecular bone thickness (Tb.Th), and trabecular bone separation (Tb.Sp) of distal femur from young and aged mice determined by micro-CT (*n* = 12). (**B**) Representative micro-CT 3D reconstruction images. Scale bar: 500 μm. (**C**) Cortical bone area fraction (Ct.Ar/Tt.Ar) and cortical bone thickness (Ct.Th) of femur midshaft from young and aged mice determined by micro-CT (*n* = 12). (**D**) Representative micro-CT 3D reconstruction images. Scale bar: 500 μm. (**E**) Double calcein labeling images and bone formation rate (BFR) quantification in femur of young and aged mice (*n* = 12). Scale bar: 50 μm. (**F**) Quantification of maximum load and stiffness in 3-point bending test in young and aged mouse femurs (*n* = 12). (**G**) Quantification of adipocyte area (Ad.Ar/Ma.Ar) and number (Ad.N/Ma.Ar) on the basis of Oil Red O staining of femur of young and aged mice (*n* = 12). Scale bar: 500 μm. (**H**) Alizarin red (AR) staining and quantification of in vitro BMSC culture from young and aged mice. Scale bar: 20 μm. (**I**) mRNA expression of osteogenesis markers *Runx2*, *Opn*, *Bsp*, and *Ocn* following induction of BMSCs from young and aged mice to undergo osteogenic differentiation (*n* = 6). (**J**) Oil Red O staining images of in vitro BMSC culture from young and aged mice when induced to undergo adipogenic differentiation. Scale bar: 10 μm. (**K**) mRNA expression of adipogenesis markers (*Pparγ*, *C/ebpα*, *aP2*, and *Glut4*) determined when BMSCs from young and aged mice were induced to undergo adipogenic differentiation (*n* = 6). All data were obtained from 3 independent experiments. The images and numerical data are representative. Data are presented as mean ± SD; Student’s *t* test; *P < 0.05, **P < 0.01, ***P < 0.001.

Femurs were harvested from young and aged mice; those from aged mice were longer than those from young mice (Supplemental Figure 1A; supplemental material available online with this article; https://doi.org/10.1172/jci.insight.159107DS1). Micro-CT revealed a significant decrease in trabecular bone volume fraction (BV/TV), trabecular bone number (Tb.N), and trabecular bone thickness (Tb.Th) and a significant increase in trabecular bone separation (Tb.Sp) in the distal femur of aged mice compared with young mice (Figure 1A). Micro-CT 3D reconstruction images revealed the same pattern (Figure 1B). A decrease in cortical bone area fraction (Ct.Ar/Tt.Ar) and thickness (Ct.Th) was observed in the femur midshaft of aged mice compared with young mice (Figure 1, C and D). Micro-CT data revealed significant bone loss in aged mice.

Histomorphometric analysis revealed a decrease in osteoblast surface (Ob.S/bone surface [BS]) and number (Ob.N/bone perimeter [B.Pm]) (Supplemental Figure 1B), and bone formation rate (BFR) (Figure 1E), as well as an increase in osteoclast surface (Oc.S/BS) and number (Oc.N/B.Pm) (Supplemental Figure 1C), in aged mice, suggesting bone formation attenuation and bone resorption enhancement. Three-point bending tests revealed that the femur maximum load and stiffness were lower in aged mice compared with young mice (Figure 1F).

Bone and marrow fat are intrinsically linked, as both arise from a common progenitor in the marrow and can be modulated by intrinsic and extrinsic factors. Histomorphometric analysis, based on Oil Red O staining, revealed a significant increase in adipocyte area (Ad.Ar/Ma.Ar) and number (Ad.N/Ma.Ar) in the bone marrow cavity of the distal femur of aged mice (Figure 1G).

Flow cytometry results showed significantly lower proportions of CD73-positive and Sca1-positive cells in the bone marrow of aged mice compared with young mice (Supplemental Figure 2), indicating a decrease in the proportion of progenitor populations in bone marrow of aged mice.

BMSCs were induced to undergo osteogenic differentiation in vitro. Alizarin red (AR) staining and quantification revealed lower calcium deposition in aged BMSCs than young BMSCs (Figure 1H). Quantitative reverse transcription PCR (qRT-PCR) analysis revealed weaker activation of osteogenesis marker genes, including *Runx2*, *Opn*, bone sialoprotein (*Bsp*), and *Ocn* mRNA expression, in aged BMSCs compared with young BMSCs (Figure 1I). Moreover, BMSCs were induced to undergo adipogenic differentiation in vitro. Oil Red O staining revealed stronger fat droplet formation in aged BMSCs than young BMSCs (Figure 1J). Subsequent qRT-PCR analysis revealed stronger activation of the adipogenesis marker genes peroxisome proliferator–activated receptor γ (*Pparγ*), *C/ebpα*, aP2, and glucose transporter 4 (*Glut4*) in aged BMSCs compared with young BMSCs (Figure 1K).

### CCL3 accumulates in the bone marrow of aged mice.

**Figure 2:**
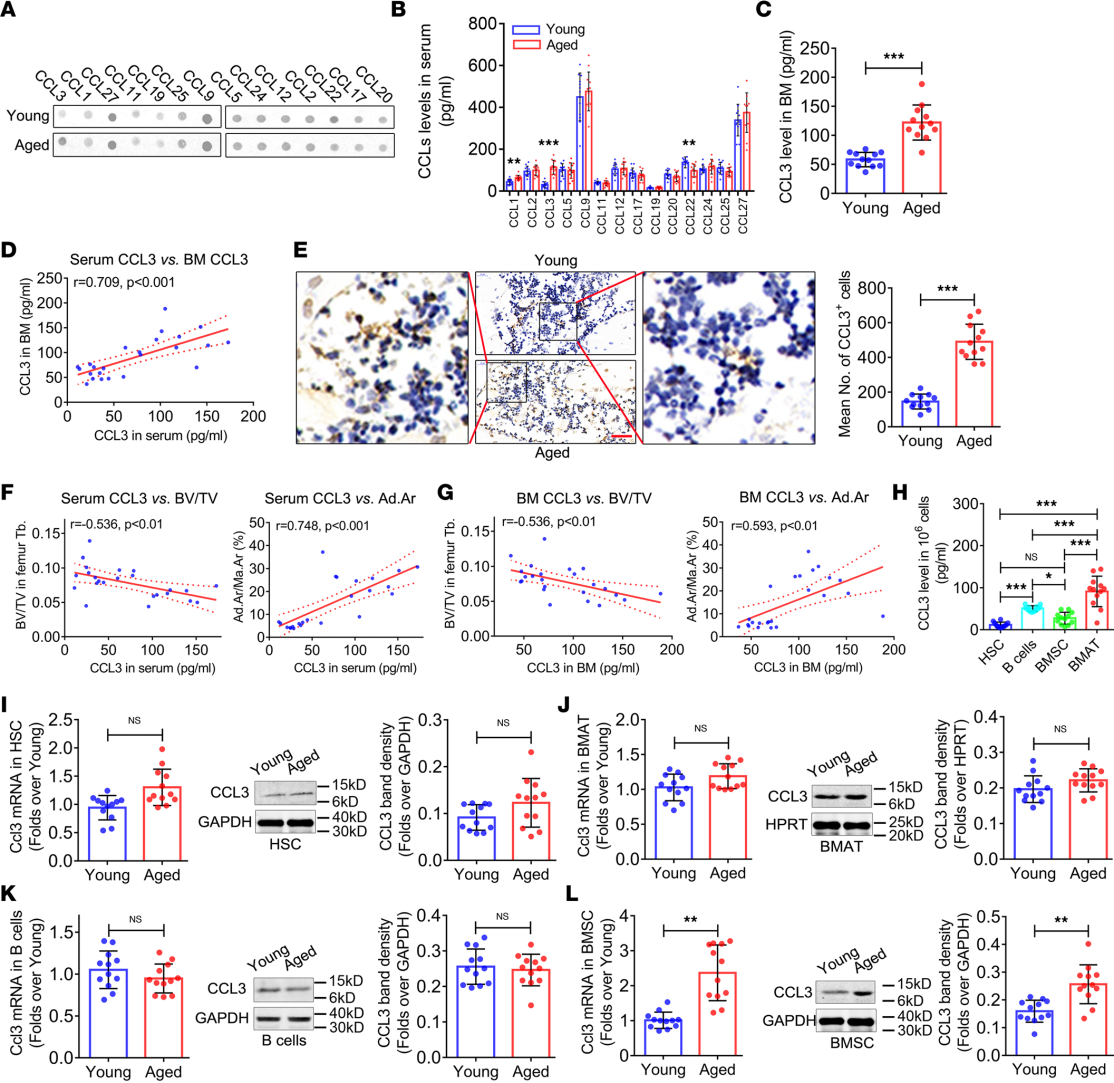
CCL3 accumulates in the bone marrow of aged mice. (**A**) Representative image of antibody array against CCL family members in peripheral blood serum from young and aged mice. (**B**) Levels of CCL family members in peripheral blood serum from young and aged mice (*n* = 12). (**C**) CCL3 level in femur bone marrow of young and aged mice (*n* = 12). (**D**) Correlation analysis of serum with bone marrow CCL3 levels. (**E**) CCL3 expression in femur bone marrow of young and aged mice and quantification of CCL3^+^ cells. Scale bars: 40 μm (original photo), 10 μm (zoomed-in photo). (**F**) Correlation analysis of serum CCL3 levels with BV/TV and Ad.Ar/Ma.Ar of femur. (**G**) Correlation analysis of CCL3 levels in bone marrow with BV/TV and Ad.Ar/Ma.Ar. (**H**) CCL3 secretion levels in in vitro 24-hour culture supernatant of HSCs, B cells, BMSCs, and BMAT (*n* = 12). CCL3 mRNA and protein expression in HSCs (**I**), BMAT (**J**), B cells (**K**), and BMSCs (**L**) from young and aged mice (*n* = 12). All data were obtained from 3 independent experiments. The images and numerical data are representative. Data are presented as mean ± SD; 1-way ANOVA in **H**; Pearson’s correlation was used to obtain r values in **D**, **F**, and **G**; Student’s *t* test was used in the other experiments. *P < 0.05, **P < 0.01, ***P < 0.001.

Chemokines, divided into the 2 major subgroups, CCL and CXCL, are essential factors involved in the regulation of cell viability, proliferation, differentiation, and migration (14). Having observed increased bone loss and bone marrow adiposity in aged mice, we next investigated the role of CCL family members in skeletal aging. An antibody array screening the secretion levels of 14 CCL family members (CCL1, 2, 3, 5, 9, 11, 12, 17, 19, 20, 22, 24, 25, and 27) in peripheral blood serum from young and aged mice (Figure 2A) revealed that CCL1, CCL3, and CCL22 exhibited significant changes in secretion levels with aging (Figure 2B). Most notably, CCL3 showed an over 2-fold increase in aged mice compared with young mice (Figure 2B).

Chemokines can be constitutively secreted by hematopoietic cells and BMSCs (14). Thus, we further investigated CCL3 levels in femur bone marrow plasma. ELISA results (Figure 2C) revealed a higher CCL3 level in the bone marrow of aged mice compared with young mice, which was positively associated with CCL3 levels in serum (Figure 2D). The immunohistochemistry results also exhibited a higher CCL3 protein expression level in the bone marrow of aged mice (Figure 2E).

We explored the correlation between serum and bone marrow CCL3 levels with bone mass and bone marrow adipose tissue (BMAT) quantities in the femur. Serum CCL3 level was negatively associated with femur BV/TV and positively associated with the Ad.Ar/Ma.Ar of femur bone marrow (Figure 2F). Correlation analysis of CCL3 levels in the bone marrow with BV/TV and Ad.Ar/Ma.Ar of the femur showed the same pattern (Figure 2G).

To clarify the source of high-level CCL3 in the bone marrow of aged mice, HSCs, BMSCs, B cells, and BMAT were isolated from aged mouse bone marrow and cultured in vitro. ELISA results showed that among the 4 cell types, BMAT exhibited the strongest CCL3 secretion ability, followed by B cells, BMSCs, and HSCs (Figure 2H). Furthermore, CCL3 mRNA and protein expression levels did not differ significantly in the HSCs (Figure 2I), B cells (Figure 2J), or BMAT (Figure 2K) between aged and young mice, while those in BMSCs were upregulated in aged mice compared with young mice (Figure 2L).

### Deletion of CCL3 increases in vivo bone mass and decreases bone marrow adiposity of aged mice.

To determine if CCL3 contributes to the observed increase in bone loss and bone marrow adiposity of aged mice, we used *Ccl3*^–/–^ mice. Immunohistochemical analysis and ELISA demonstrated the absence of CCL3 expression in the bone marrow and serum of *Ccl3*^–/–^ mice, while CCL3 was expressed in the bone marrow of both young (Supplemental Figure 3, A and B) and aged (Supplemental Figure 3, C and D) WT mice.

**Figure 3:**
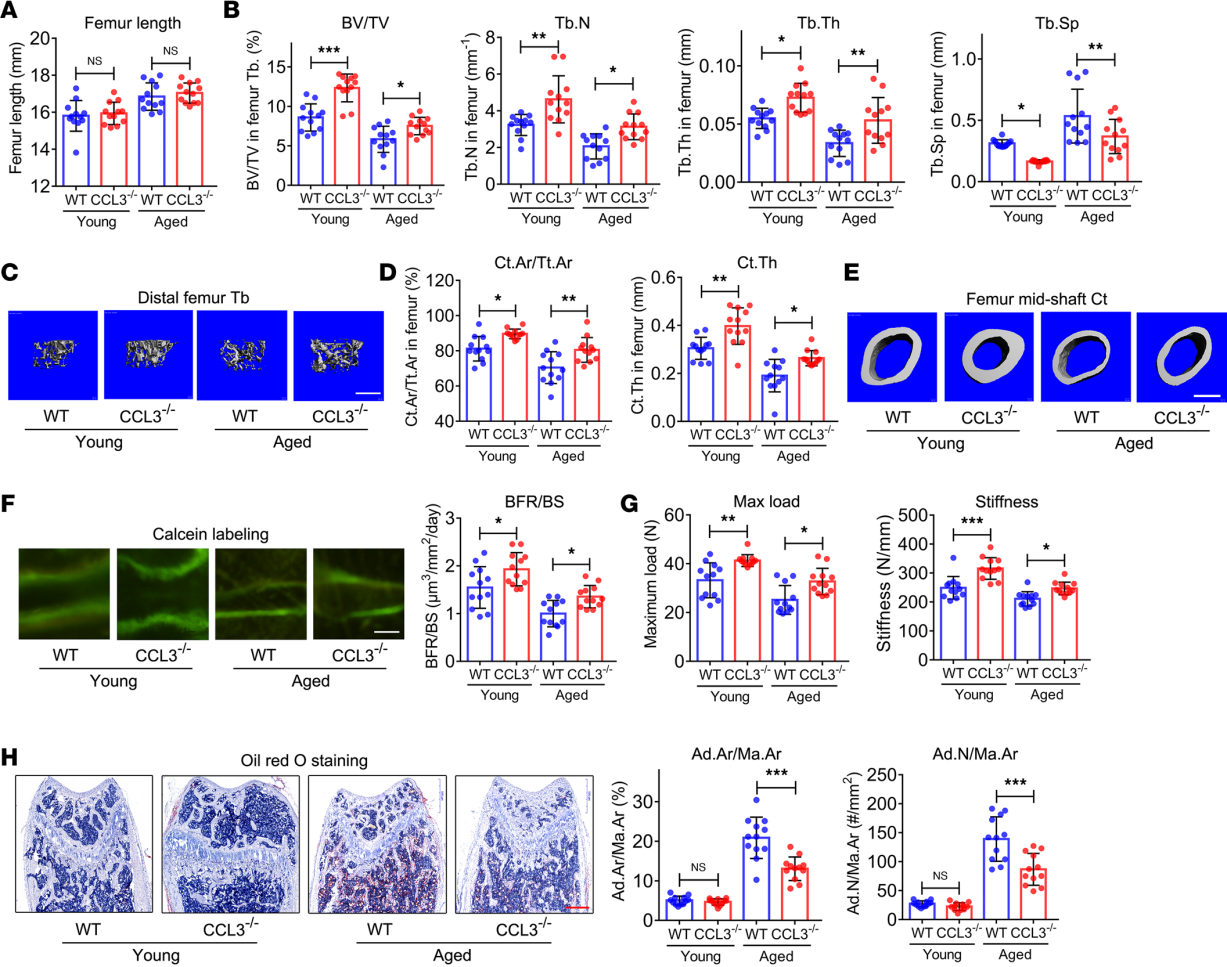
Deletion of CCL3 counteracts in vivo bone loss and bone marrow adiposity of aged mice. (**A**) Femur length of young and aged wild-type (WT) and *Ccl3^–/–^* mice (*n* = 12). (**B**) BV/TV, Tb.N, Tb.Th, and Tb.Sp of distal femur from young and aged WT and *Ccl3^–/–^* mice (*n* = 12). (**C**) Representative micro-CT 3D reconstruction images. Scale bar: 500 μm. (**D**) Ct.Ar/Tt.Ar and Ct.Th of femur midshaft from young and aged WT and *Ccl3^–/–^* mice (*n* = 12). (**E**) Representative micro-CT 3D reconstruction images. Scale bar: 500 μm. (**F**) Double calcein labeling images and BFR quantification in femur of young and aged WT and *Ccl3^–/–^* mice (*n* = 12). Scale bar: 50 μm. (**G**) Quantification of maximum load and stiffness in 3-point bending test in femur of young and aged WT and *Ccl3^–/–^* mice (*n* = 12). (**H**) Quantification of Ad.Ar/Ma.Ar and Ad.N/Ma.Ar on the basis of Oil Red O staining of young and aged WT and *Ccl3^–/–^* mice femurs (*n* = 12). Scale bar: 500 μm. All data were obtained from 3 independent experiments. The images and numerical data are representative. Data are presented as mean ± SD; 2-way ANOVA. *P < 0.05, **P < 0.01, ***P < 0.001.

Femurs were collected from young and aged *Ccl3*^–/–^ and WT mice, showing no significant differences in length between conditions with and without *Ccl3* deletion (Figure 3A). However, micro-CT scanning showed a significant increase in BV/TV, Tb.N, and Tb.Th, as well as a significant decrease in Tb.Sp, in the distal femur of *Ccl3*^–/–^ mice compared with WT mice, regardless of age (Figure 3B). Micro-CT 3D reconstruction images showed the same pattern (Figure 3C). Similarly, an increase was observed in Ct.Ar/Tt.Ar and Ct.Th in the femur midshaft of *Ccl3^–/–^* mice compared with WT mice (Figure 3, D and E). Histomorphology analysis further revealed increased Ob.S/BS, Ob.N/B.Pm (Supplemental Figure 3E), and BFR (Figure 3F) and decreased Oc.S/BS and Oc.N/B.Pm (Supplemental Figure 3F) in *Ccl3*^–/–^ mice, suggesting enhanced bone formation and attenuated bone resorption in *Ccl3^–/–^* mice compared with WT mice. Three-point bending tests showed that the maximum load and stiffness of femurs were improved in *Ccl3^–/–^* mice compared with WT mice (Figure 3G), while histomorphometric analysis, based on Oil Red O staining, revealed a significant decrease in Ad.Ar/Ma.Ar and Ad.N/Ma.Ar in the bone marrow cavity of distal femur of aged *Ccl3^–/–^* mice (Figure 3H) compared with aged WT mice.

In addition, no significant differences were exhibited between *Ccl3^–/–^* and WT mice in body weight (Supplemental Figure 4A), abdominal fat pad weight (Supplemental Figure 4B), serum levels of lipid metabolism markers (low-density lipoprotein, high-density lipoprotein, and triglyceride; Supplemental Figure 4C), serum levels of inflammatory factors (IL-1β, IL-2, IL-4, IL-5, IL-6, and IL-9; Supplemental Figure 4D), or serum levels of endocrine function markers (glucocorticoid, aldosterone, and insulin; Supplemental Figure 4E).

### CCL3 improves BMSCs’ adipogenic differentiation potential via STAT3-mediated C/EBPα activation.

**Figure 4:**
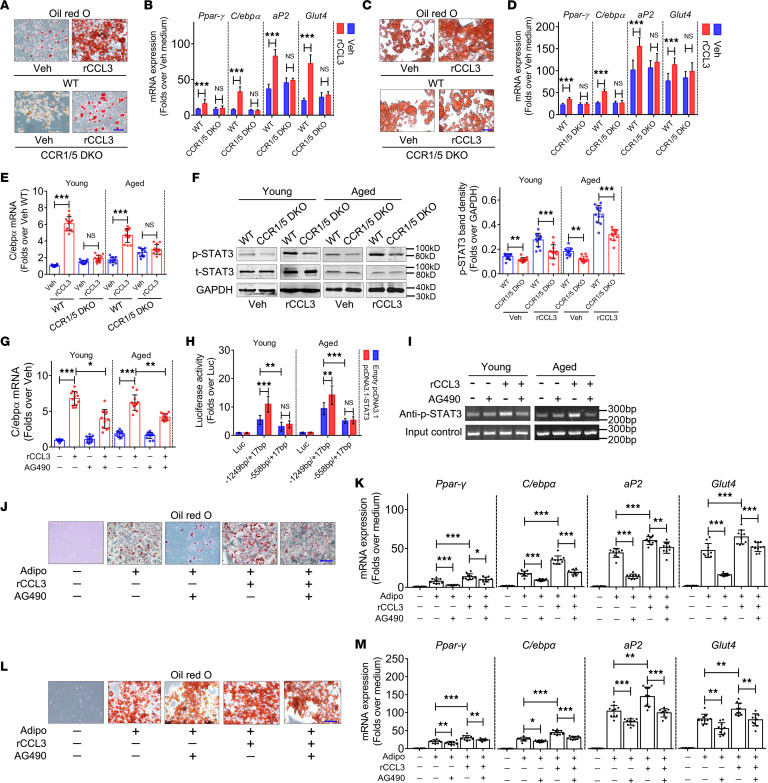
CCL3 improves the adipogenic differentiation potential of BMSCs via STAT3-mediated C/EBPα activation. (**A**) Oil Red O staining of BMSCs (young) cultured with rCCL3. Scale bar: 10 μm. (**B**) mRNA expression of *Pparg*, *C/ebpa*, *aP2*, and *Glut4* when BMSCs (young) undergo adipogenic differentiation with rCCL3 treatment (*n* = 12). (**C**) Oil Red O staining of BMSCs (aged) cultured with rCCL3. Scale bar: 10 μm. (**D**) mRNA expression of *Pparg*, *C/ebpa*, *aP2*, and *Glut4* when BMSCs (aged) undergo adipogenic differentiation with rCCL3 (*n* = 12). (**E**) *C/ebpa* mRNA expression in young and aged BMSCs with rCCL3 treatment (*n* = 12). (**F**) Phosphorylated and total STAT3 protein expression in BMSCs with rCCL3 treatment (*n* = 12). (**G**) *C/ebpa* mRNA expression in response to rCCL3 and/or AG490 treatment in BMSCs (*n* = 12). (**H**) Luciferase activity of C/EBPα promoter deletion mutant–driven luciferase reporter gene vectors, including Luc (empty vector as negative control), –1,249 bp/+17 bp (containing putative STAT3 binding site) and –558 bp/+17 bp (putative STAT3 binding site deleted), with STAT3 overexpression, in BMSCs treated with rCCL3 for 48 hours (*n* = 12). (**I**) ChIP assay using STAT3 antibody against putative STAT3 binding site within C/EBPα promoter in BMSCs treated with rCCL3 and/or AG490. (**J**) Oil Red O staining of BMSCs (young) after undergoing adipogenic differentiation with rCCL3 and/or AG490 treatment. (**K**) mRNA expression of *Pparg*, *C/ebpa*, *aP2*, and *Glut4* when BMSCs (young) undergo adipogenic differentiation with rCCL3 and/or AG490 treatment (*n* = 10). (**L**) Oil Red O staining of BMSCs (aged) after undergoing adipogenic differentiation with rCCL3 and/or AG490 treatment. (**M**) mRNA expression of *Pparg*, *C/ebpa*, *aP2*, and *Glut4* when BMSCs (aged) undergo adipogenic differentiation with rCCL3 and/or AG490 treatment (*n* = 10). Hprt was used as internal control. All data were obtained from 3 independent experiments. Statistics, 2-way ANOVA. *P < 0.05, **P < 0.01, ***P < 0.001. Scale bar: 10 μm (**J** and **L**).

CCL3 binds to CCR1 and CCR5 receptors (14). BMSCs, from young and aged *Ccr1/Ccr5* double-knockout (DKO) and WT mice, were induced to undergo adipogenic differentiation in vitro. In young BMSCs from WT mice, Oil Red O staining revealed significant enhancement of fat droplet formation in response to recombinant CCL3 (rCCL3) treatment compared with vehicle (Figure 4A). qRT-PCR analysis revealed increased *Pparγ*, *C/ebpα*, *aP2*, and *Glut4* mRNA expression in response to rCCL3 treatment than vehicle (Figure 4B). However, in young BMSCs (Figure 4, A and B) and aged BMSCs (Figure 4, C and D) from *Ccr1/5* DKO mice, the enhanced fat droplet formation and activation of adipogenic differentiation markers in response to rCCL3 were prevented (Figure 4, A and B). These data suggested that CCL3 promoted BMSCs’ adipogenic differentiation via CCR1 and CCR5.

The expressions of PPARγ and C/EBPα — key transcriptional factors that control BMSCs’ adipogenic differentiation — were activated by CCL3, implying that these transcriptional factors might be CCL3 targets to improve the adipogenic differentiation potential (Figure 4, B and D). Of note, C/EBPα expression was weaker in BMSCs from young and aged *Ccr1/5* DKO mice compared with WT mice. Consequently, we focused on C/EBPα to elucidate the role of CCL3 in BMSCs’ adipogenic differentiation. Minimal rCCL3 effects were retained on the expression of adipogenic differentiation markers in young (Figure 4B) and aged (Figure 4D) *Ccr1/5* DKO mice, implying that another signaling pathway, independent of CCR1 and CCR5, may influence the biological functions of CCL3.

When rCCL3 was added to the medium of young and aged BMSCs from WT mice, C/EBPα mRNA expression was remarkably upregulated (Figure 4E). However, C/EBPα mRNA expression activation by CCL3 was canceled in young and aged BMSCs from *Ccr1/5* DKO mice (Figure 4E).

STAT3, important downstream effector of CCL3 (21), was phosphorylated in young and aged BMSCs from WT mice in response to rCCL3 treatment (Figure 4F). In contrast, rCCL3 failed to enhance STAT3 phosphorylation in young and aged BMSCs from *Ccr1/5* DKO mice compared to WT mice (Figure 4F). Moreover, the rCCL3-induced upregulation of C/EBPα expression was attenuated upon STAT3 inhibition by its specific inhibitor AG490 (Figure 4G), suggesting that STAT3 was involved in C/EBPα expression activation by CCL3.

The C/EBPα promoter (–1,249 to +17 bp) was analyzed using JASPAR (http://jaspar.genereg.net/) to screen transcription factor binding sites, revealing a putative STAT3 binding site (–621 to –612 bp; Supplemental Figure 5). We amplified the mouse C/EBPα promoter –1,249 to +17 bp and generated a deletion mutant –558 bp/+17 bp, in which the putative STAT3 binding site was deleted. The various C/EBPα promoter fragments were subcloned into luciferase reporter gene vectors (Supplemental Figure 6), and a transient reporter assay was performed in young and aged BMSCs from WT mice following rCCL3 treatment. The luciferase activity of the complete C/EBPα promoter–driven construct exhibited significant increases compared with the empty luciferase vector (Figure 4H). However, a marked decrease was observed in the activity of the STAT3 binding site deletion mutant–driven construct compared with the complete promoter–driven construct (Figure 4H). To confirm the role of the STAT3 binding site in C/EBPα expression, we constructed a plasmid vector to overexpress STAT3 and cotransfected it with the C/EBPα promoter–driven constructs, with or without putative STAT3 binding sites, into young and aged BMSCs from WT mice. The complete C/EBPα promoter–driven construct activity was remarkably enhanced in response to STAT3 overexpression (Figure 4H). In contrast, STAT3 overexpression failed to enhance the activity of the STAT3 binding site deletion mutant–driven construct (Figure 4H). Further, we evaluated the binding of STAT3 to the –621 to –612 bp region of the C/EBPα promoter in young and aged BMSCs from WT mice in response to rCCL3 and/or AG490 treatment. ChIP results validated the binding of STAT3 to the C/EBPα promoter (Figure 4I). rCCL3 enhanced STAT3 binding to the C/EBPα promoter, which was lowered by AG490 (Figure 4I).

BMSCs from young and aged mice were induced to undergo adipogenic differentiation in vitro in response to rCCL3 and/or AG490 treatment. Oil Red O staining revealed significant enhancement of fat droplet formation of young BMSCs in response to rCCL3 treatment, which was lowered by AG490 (Figure 4J). These results were validated by qRT-PCR (Figure 4K). Fat droplet formation (Figure 4L) and expression of adipogenic differentiation markers (Figure 4M) in response to rCCL3 and/or AG490 exhibited the same patterns in aged BMSCs as young BMSCs.

### DNA hypomethylation in CCL3 promoter region facilitates C/EBPα-activated CCL3 expression during adipogenic differentiation of BMSCs.

**Figure 5:**
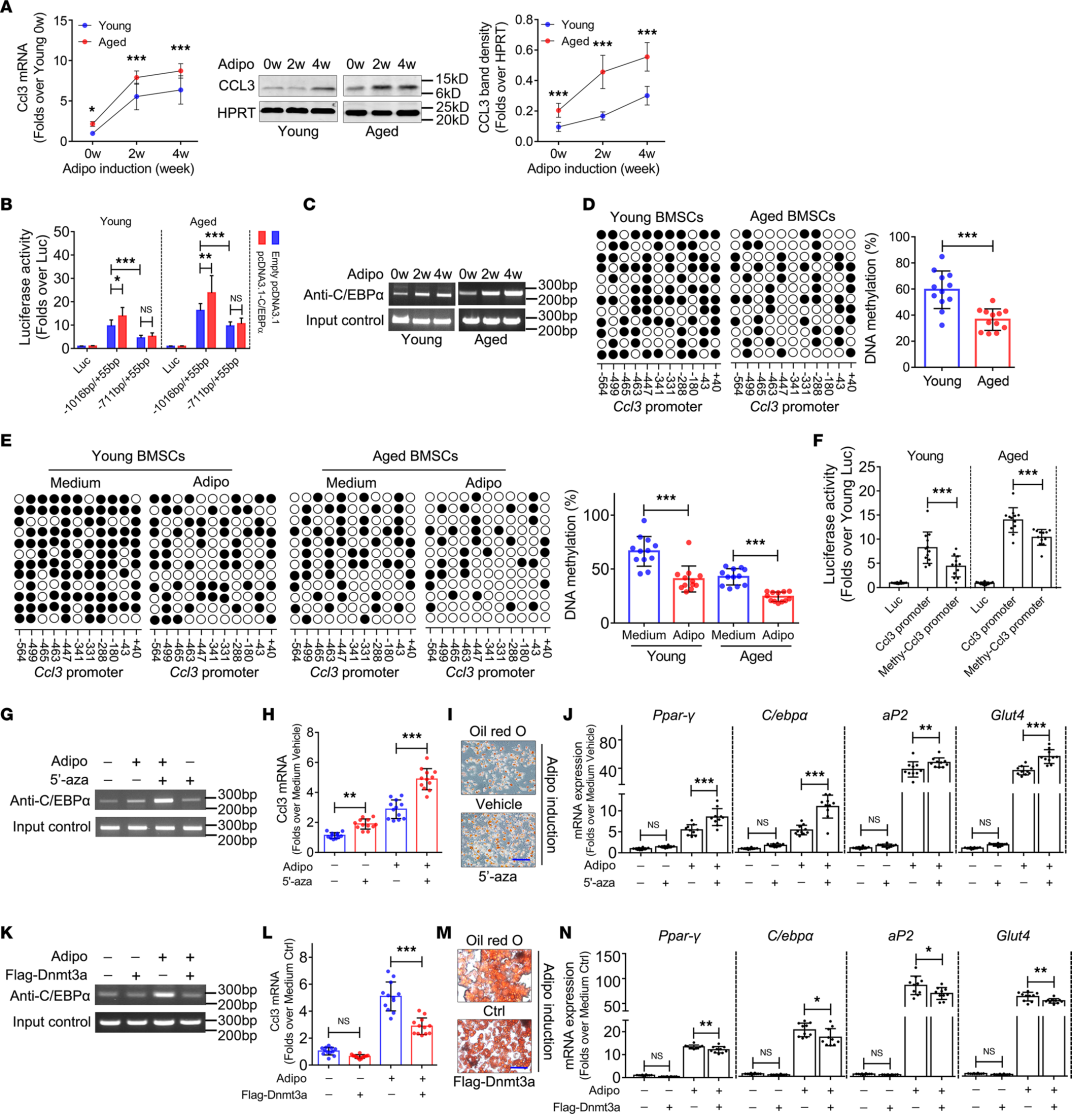
DNA hypomethylation in the CCL3 promoter region facilitates C/EBPα-activated CCL3 expression during adipogenic differentiation of BMSCs. (**A**) CCL3 mRNA and protein expression when BMSCs undergo adipogenic differentiation (*n* = 12). (**B**) Luciferase activity of CCL3 promoter deletion mutant–driven luciferase reporter gene vector in BMSCs undergoing adipogenic differentiation (*n* = 12). (**C**) ChIP assay using C/EBPα antibody against the putative C/EBPα binding site within CCL3 promoter when BMSCs undergo adipogenic differentiation (*n* = 12). (**D**) DNA methylation percentage of 11 CG sites within proximal CCL3 promoter in BMSCs (*n* = 12). (**E**) DNA methylation percentage of 11 CG sites within proximal CCL3 promoter in BMSCs with adipogenesis induction (*n* = 12). (**F**) Luciferase activity of methylated and unmethylated CCL3 promoter–driven luciferase reporter gene vector in BMSCs undergo adipogenic differentiation (*n* = 12). (**G**) ChIP assay using C/EBPα antibody against the putative C/EBPα binding site within the CCL3 promoter in BMSCs treated with 5′-aza. (**H**) *Ccl3* mRNA expression in response to 5′-aza in young BMSCs (*n* = 12). (**I**) Oil Red O staining of BMSCs undergoing adipogenic differentiation with 5′-aza treatment. (**J**) mRNA expression when BMSCs undergo adipogenic differentiation with 5′-aza treatment (*n* = 10). (**K**) ChIP assay using C/EBPα antibody against the putative C/EBPα binding site within the CCL3 promoter of aged BMSCs when Dnmt3a was overexpressed. (**L**) *Ccl3* mRNA expression in response to Dnmt3a overexpression in aged BMSCs (*n* = 12). (M) Oil Red O staining of aged BMSCs undergoing adipogenic differentiation with Dnmt3a overexpression. (**N**) mRNA expression of Pparγ, C/ebpα, aP2, and Glut4 when aged BMSCs undergo adipogenic differentiation with Dnmt3a overexpression (*n* = 10). All data were obtained from 3 independent experiments. Statistics, Student’s *t* test (**D**); 1-way ANOVA (**F**); 2-way ANOVA (**A**, **B**, **E**, **H**, **J**, **L**, and **N**). *P < 0.05, **P < 0.01, ***P < 0.001. Scale bar: 10 μm (**I** and **M**).

We subsequently analyzed the molecular mechanisms underlying CCL3 accumulation in the bone marrow of aged mice. First, the mRNA and protein expression of CCL3 were investigated during in vitro adipogenic differentiation of young and aged BMSCs from WT mice. qRT-PCR and Western blot analysis revealed a progressive upregulation of CCL3 expression during adipogenic differentiation (Figure 5A). The CCL3 promoter (–1,016 to +55 bp) was screened to predict transcriptional factor binding sites, in which a putative C/EBPα binding site (–854 to –843 bp) was observed (Supplemental Figure 7). The mouse CCL3 promoter (–1,016 to +55 bp) was amplified, and a deletion mutant (–711 bp/+55 bp) was generated in which the putative C/EBPα binding site was deleted. The various CCL3 promoter fragments were subcloned into a luciferase reporter gene vector (Supplemental Figure 8). Subsequently, a transient reporter assay was performed in young and aged BMSCs from WT mice following adipogenic induction. The luciferase activity of the complete CCL3 promoter–driven construct showed marked increases compared with the empty luciferase vector (Figure 5B). However, a decrease was observed in the activity of the C/EBPα binding site deletion mutant–driven construct compared with the complete promoter–driven construct (Figure 5B). We then constructed a plasmid vector to overexpress C/EBPα and cotransfected it with the CCL3 promoter–driven constructs, with or without the putative C/EBPα binding site, into young and aged BMSCs from WT mice. The activity of the complete CCL3 promoter–driven construct was enhanced in response to C/EBPα overexpression (Figure 5B). In contrast, C/EBPα overexpression failed to enhance the activity of the C/EBPα binding site deletion mutant–driven construct (Figure 5B). Further, we evaluated the binding of C/EBPα to the –854 to –843 bp region of the CCL3 promoter in young and aged BMSCs from WT mice upon induction of adipogenic differentiation. ChIP results validated the binding of C/EBPα to the CCL3 promoter (Figure 5C). Moreover, C/EBPα binding to the CCL3 promoter increased progressively from weeks 0–4 of BMSCs’ adipogenic differentiation (Figure 5C).

The promoter DNA methylation status is closely related to transcription activation. The CCL3 promoter contains 11 CpG sites (i.e., potential DNA methylation targets) around the transcription start site (Supplemental Figures 7 and 9). The methylation status of these 11 CpG sites was investigated in BMSCs from young and aged mice. DNA hypomethylation within the CCL3 promoter was revealed in BMSCs from aged mice compared with young mice (Figure 5D). Further, the methylation status of these 11 CpG sites was investigated during adipogenic differentiation of young and aged BMSCs from WT mice. Significant DNA hypomethylation was observed in BMSCs induced to undergo adipogenesis compared with noninduced cells (Figure 5E). M.*Sss*I was utilized to drive in vitro methylation of the CCL3 promoter, which was subcloned into the luciferase reporter gene vector. Transient reporter assays were then performed in young and aged BMSCs from WT mice. CCL3 promoter methylation led to reduced luciferase activity (Figure 5F).

Addition of the DNA methyltransferase inhibitor 5′-aza — known to trigger DNA hypomethylation — to the medium of young BMSCs caused reduced DNA methylation of the CCL3 promoter (Supplemental Figure 10A). Meanwhile, ChIP results showed that C/EBPα binding to the CCL3 promoter was enhanced by 5′-aza (Figure 5G). *Ccl3* mRNA expression was markedly upregulated (Figure 5H). Further, fat droplet formation (Figure 5I) and the expression of adipogenic differentiation markers (Figure 5J) were enhanced in response to 5′-aza.

When de novo methyltransferase 3a (Dnmt3a) — responsible for adding methyl groups to DNA — was stably overexpressed in aged BMSCs from WT mice (Supplemental Figure 10B), DNA methylation in the CCL3 promoter was increased (Supplemental Figure 10C). Meanwhile, ChIP results showed that C/EBPα binding to the CCL3 promoter was inhibited by Dnmt3a overexpression (Figure 5K) and *Ccl3* mRNA expression was downregulated (Figure 5L). Further, fat droplet formation (Figure 5M) and the expression of adipogenic differentiation markers (Figure 5N) were blocked in response to Dnmt3a overexpression.

### CCL3 inhibits BMSCs’ osteogenic differentiation potential by ERK-mediated DKK1 upregulation.

The bone resorption activity of mice was slightly lowered in response to in vivo *Ccl3* deletion (Supplemental Figure 3F), implying a positive role for CCL3 in osteoclast activity, which agrees with several previous studies (13, 22, 23). Bone marrow monocytes (BMMs), from young and aged mice and WT, *Ccl3^–/–^*, and *Ccr1/5* DKO mice, were induced to undergo osteoclastic differentiation in vitro. ELISA showed a progressive elevation of CCL3 secretion with osteoclast differentiation of BMMs from young and aged mice (Supplemental Figure 11A). No significant difference was observed in CCL3 secretion from BMMs between young and aged mice (Supplemental Figure 11A). Tartrate-resistant acid phosphatase staining and quantification and qRT-PCR revealed: i) No difference in osteoclast differentiation potential was observed between young and aged mice (Supplemental Figure 11, B and C). ii) Osteoclast differentiation was blocked in response to neutralizing antibody against CCL3 (Supplemental Figure 12, A and B). iii) Osteoclast differentiation was markedly lowered in BMMs from *Ccl3^–/–^* mice compared with WT mice, which was recovered by rCCL3 (Supplemental Figure 12, C and D). iv) Osteoclast differentiation activated by rCCL3 was abrogated in BMMs from *Ccr1/5* DKO mice (Supplemental Figure 12, E and F). These results suggested that CCL3 utilized both CCR1 and CCR5 to induce osteoclast differentiation, as reported previously (24).

**Figure 6:**
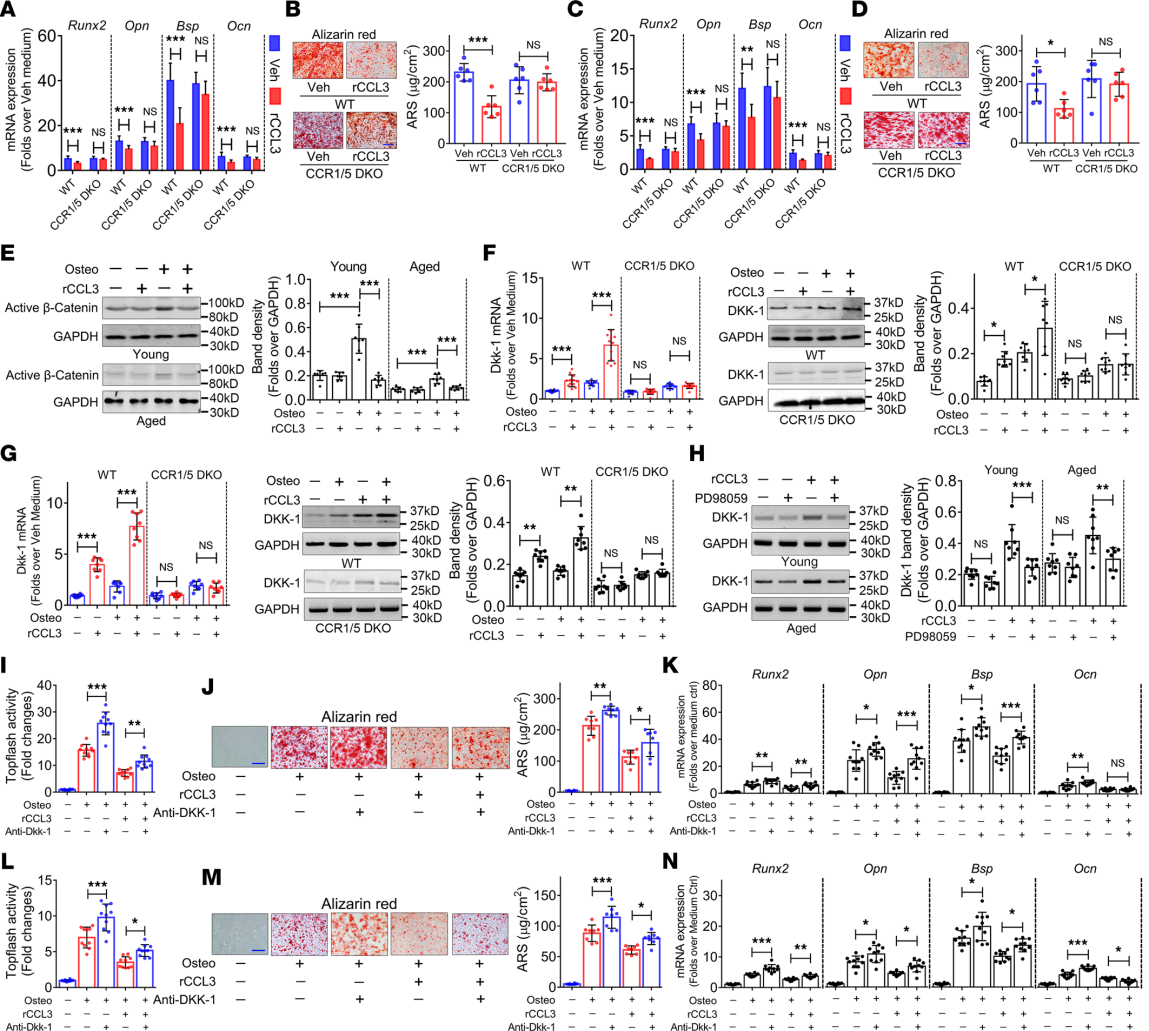
CCL3 inhibits osteogenic differentiation potential of BMSCs by ERK-mediated *Dkk1* upregulation. (**A**) mRNA expression of *Runx2*, *Opn*, *Bsp*, and *Ocn* when BMSCs from young mice undergo osteogenic differentiation with rCCL3 treatment (*n* = 10). (**B**) AR staining and quantification of BMSCs cultured from young mice with rCCL3 treatment. Scale bar: 20 μm. (**C**) mRNA expression of *Runx2*, *Opn*, *Bsp*, and *Ocn* when BMSCs from aged mice undergo osteogenic differentiation with rCCL3 treatment (*n* = 10). (**D**) AR staining and quantification of BMSCs cultured from aged mice with rCCL3 treatment. Scale bar: 20 μm. (**E**) Nonphosphorylated (active) β-catenin protein expression when BMSCs undergo osteogenic differentiation with rCCL3 treatment. (**F**) DKK1 mRNA and protein expression when BMSCs undergo osteogenic differentiation with rCCL3 treatment. (**G**) DKK1 mRNA and protein expression when BMSCs undergo osteogenic differentiation with rCCL3 treatment. (**H**) DKK1 protein expression in BMSCs with rCCL3 and/or PD98059 treatment. (**I**) Active β-catenin activity when BMSCs from young mice undergo osteogenic differentiation with rCCL3 and/or neu anti-DKK1 treatment (*n* = 10). (**J**) AR staining and quantification of BMSCs cultured from young mice when undergoing osteogenic differentiation with rCCL3 and/or neu anti-DKK1 treatment. (**K**) mRNA expression of *Runx2*, *Opn*, *Bsp*, and *Ocn* when BMSCs from young mice undergoing osteogenic differentiation with rCCL3 and/or neu anti-DKK1 treatment (*n* = 10). (**L**) Active β-catenin activity when BMSCs from aged mice undergo osteogenic differentiation with rCCL3 and/or neu anti-DKK1 treatment (*n* = 10). (**M**) AR staining and quantification of BMSCs cultured from aged mice when undergoing osteogenic differentiation with rCCL3 and/or neu anti-DKK1 treatment. (**N**) mRNA expression of *Runx2*, *Opn*, *Bsp*, and *Ocn* when BMSCs from aged mice undergo osteogenic differentiation with rCCL3 and/or neu anti-DKK1 treatment (*n* = 10). All data were obtained from 3 independent experiments. Statistics, 2-way ANOVA. *P < 0.05, **P < 0.01, ***P < 0.001. Scale bar: 40 μm (**J** and **M**).

Next, BMSCs from young and aged *Ccr1/5* DKO and WT mice were induced to undergo osteogenic differentiation in vitro. In young BMSCs from WT mice, qRT-PCR analysis revealed weaker activation of *Runx2*, *Opn*, *Bsp*, and *Ocn* mRNA expression in response to rCCL3 treatment than vehicle (Figure 6A). AR staining and quantification revealed significantly reduced calcium deposition in response to rCCL3 treatment compared with vehicle (Figure 6B). However, in young BMSCs from *Ccr1/5* DKO mice, attenuation of osteogenic differentiation marker activation, and calcium deposition in response to rCCL3, were rescued (Figure 6, A and B). Similarly, in aged BMSCs from *Ccr1/5* DKO and WT mice, qRT-PCR and AR staining showed that the attenuated activation of adipogenic differentiation markers and calcium deposition by rCCL3 treatment were rescued in response to *Ccr1* and *Ccr5* deletion (Figure 6, C and D). These data suggested that CCL3 inhibited BMSCs’ osteogenic differentiation via CCR1 and CCR5.

To elucidate the molecular mechanisms underlying the inhibitory effect of CCL3 on BMSCs’ osteogenic differentiation, we focused on the WNT/β-catenin pathway. qRT-PCR analysis revealed no differences in the mRNA expression of Wnt/β-catenin pathway members (*Wnt1*, *Wnt3a*, *Wnt4*, *Lrp5*, *Apc*, *Gsk3b*, *Axin2*, and *Frizzled*) between vehicle- and rCCL3-treated BMSCs (Supplemental Figure 13). However, the β-catenin gene — a key effector of the WNT/β-catenin pathway — was inactivated by CCL3, regardless of whether young or aged BMSCs were induced to undergo osteogenic differentiation (Figure 6E). Further, the mRNA and protein expression of DKK1 — an inhibitor of the WNT/β-catenin signaling pathway — was upregulated by CCL3, regardless of the induction status of young (Figure 6F) or aged (Figure 6G) BMSCs from WT mice. In contrast, CCL3 failed to upregulate DKK1 in young and aged BMSCs from *Ccr1/5* DKO mice (Figure 6, F and G).

CCL3 reportedly activates ERK signaling in the regulation of osteoblast differentiation (25, 26). Here, rCCL3 activated ERK phosphorylation and DKK1 expression within 30 minutes in young and aged BMSCs (Supplemental Figure 14A). Meanwhile, addition of the ERK inhibitor, PD98059, to the medium of young and aged BMSCs prevented activation of DKK1 expression in response to rCCL3 treatment (Figure 6H). However, addition of a neutralizing DKK1 antibody to the medium of young and aged BMSCs did not inhibit ERK phosphorylation in response to rCCL3 treatment (Supplemental Figure 14B). These data suggest that ERK is upstream of DKK1.

DKK1 protein expression was upregulated in BMSCs from aged mice compared with young mice. However, weak DKK1 expression was observed in the BMSCs and bone marrow of *Ccl3^–/–^* mice (Supplemental Figure 15, A and B). In contrast, active β-catenin expression was downregulated in BMSCs of aged mice compared with young mice, whereas strong active β-catenin expression was observed in the BMSCs and bone marrow of *Ccl3^–/–^* mice (Supplemental Figure 15, C and D).

Treatment with a DKK1-neutralizing antibody prevented inactivation of β-catenin by rCCL3 in young BMSCs from WT mice (Figure 6I). Meanwhile, young BMSCs were induced to undergo osteogenic differentiation with rCCL3 and/or neutralizing (neu) anti-DKK1. AR staining (Figure 6J) and qRT-PCR (Figure 6K) showed that rCCL3 inhibited BMSCs’ osteogenic differentiation, which was rescued to a large extent by neu anti-DKK1. Similarly, in aged BMSCs, neutralization of DKK1 relieved β-catenin inactivation (Figure 6L) and rescued BMSCs’ osteogenic differentiation inhibited by rCCL3 in large part if not entirely (Figure 6, M and N).

### In vivo blockade of CCL3 ameliorates trabecular bone loss and bone marrow adiposity of aged mice.

**Figure 7:**
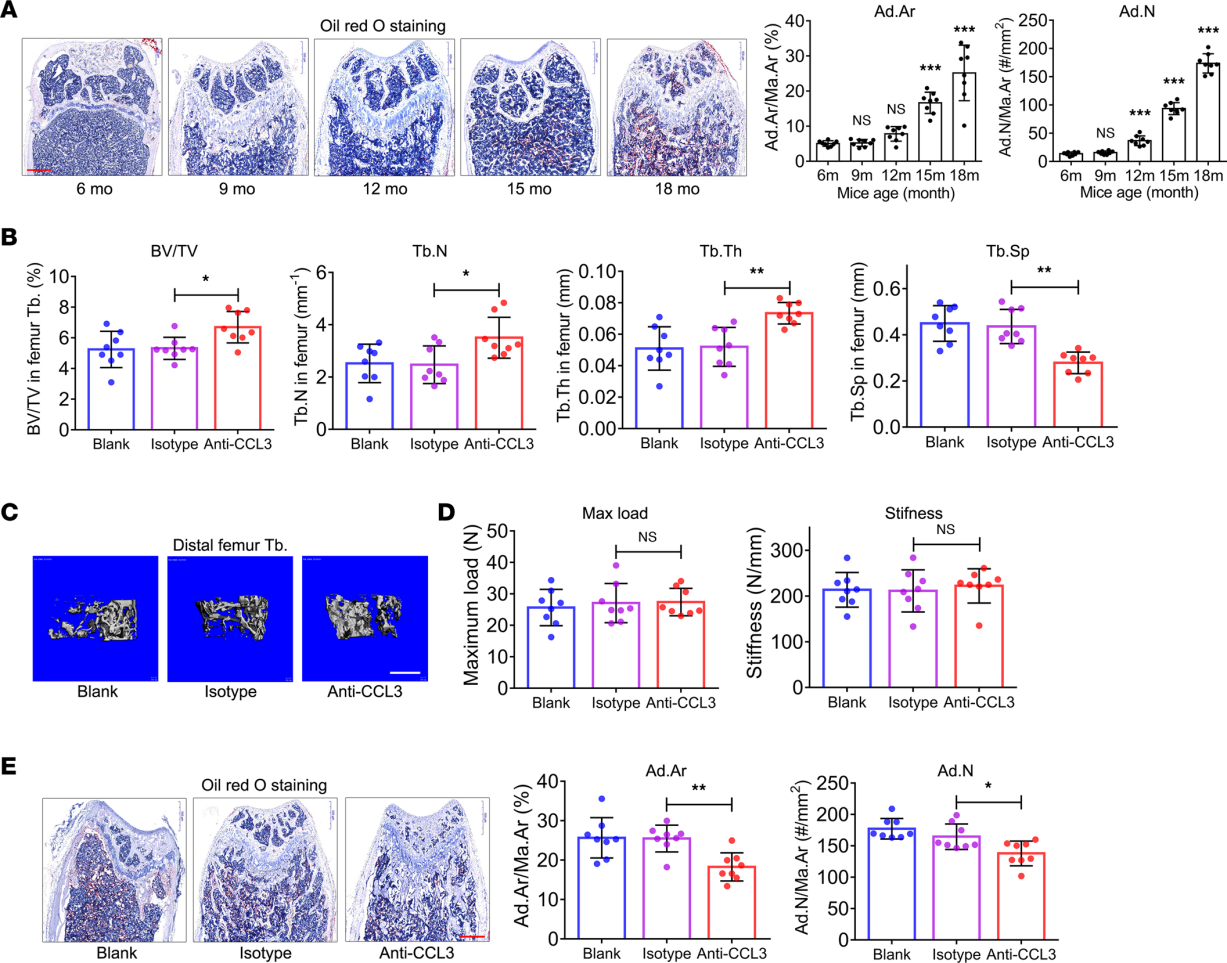
In vivo blockade of CCL3 using neutralization antibody ameliorates bone loss and bone marrow adiposity of aged mice. (**A**) Quantification of Ad.Ar/Ma.Ar and Ad.N/Ma.Ar via Oil Red O staining of femurs of 6-, 9-, 12-, 15-, and 18-month-old mice (*n* = 8). Scale bar: 500 μm. (**B**) BV/TV, Tb.N, Tb.Th, and Tb.Sp of distal femur from aged mice with neu anti-CCL3 injection (*n* = 8). (**C**) Representative micro-CT 3D reconstruction images. Scale bar: 500 μm. (**D**) Quantification of maximum load and stiffness in 3-point bending test in femur of aged mice with neu anti-CCL3 injection (*n* = 8). (**E**) Quantification of Ad.Ar/Ma.Ar and Ad.N/Ma.Ar in femurs of aged mice with neu anti-CCL3 injection (*n* = 8). Scale bar: 500 μm. All data were obtained from 3 independent experiments. The images and numerical data are representative. Data are presented as mean ± SD; 1-way ANOVA. *P < 0.05, **P < 0.01, ***P < 0.001.

To clarify the time window for bone marrow adiposity of aged mice, we collected femurs of 6-, 9-, 12-, 15-, and 18-month-old mice. Histomorphometric analysis, based on Oil Red O staining, indicated that significant bone marrow adiposity was established in the femurs of 15-month-old mice (Figure 7A). Thus, 14-month-old mice were intraperitoneally administrated a CCL3 neutralization antibody once every 10 days for 4 months (Supplemental Figure 16A). At 18 months of age, mice were euthanized for micro-CT scan and histomorphometric analysis.

Neu anti-CCL3 administration did not alter femur length (Supplemental Figure 16B); however, a significant increase occurred in BV/TV, Tb.N, and Tb.Th, and a significant decrease occurred in Tb.Sp in the distal femur of mice administered the CCL3 antibody compared with the isotype control (Figure 7B). Meanwhile, neu anti-CCL3 administration did not affect the Ct.Ar/Tt.Ar or Ct.Th of the femur (Supplemental Figure 16C). Micro-CT 3D reconstruction images showed the same pattern (Figure 7C). An increase in Ob.S/BS and Ob.N/B.Pm (Supplemental Figure 16D) and a decrease in Oc.S/BS and Oc.N/B.Pm (Supplemental Figure 16E) were observed in aged mice in response to neu anti-CCL3 administration, suggesting enhanced bone formation and attenuated bone resorption. However, 3-point bending testing showed that the maximum load and stiffness of femurs did not change in response to neu anti-CCL3 administration (Figure 7D). Moreover, a significant decrease in Ad.Ar/Ma.Ar and Ad.N/Ma.Ar was detected in the bone marrow cavity of the distal femur of aged mice in response to neu anti-CCL3 administration (Figure 7E). CCL3 neutralization antibody administration did not cause abnormal changes in the histological appearances of the heart, liver, spleen, lungs, or kidneys (Supplemental Figure 17).

Of note, although the data presented here are specific to male mice, the data from female mice were comparable. This is demonstrated in Supplemental Figure 18, which presents bone phenotype and bone marrow adiposity of aged female mice (Supplemental Figure 18, A–C), the imbalance between osteogenic (Supplemental Figure 18, D and E) and adipogenic (Supplemental Figure 18, F and G) differentiation of aged BMSCs, CCL3 accumulation in the bone marrow of aged female mice (Supplemental Figure 18, H and I), and amelioration of in vivo bone loss and bone marrow adiposity of female *Ccl*3^–/–^ mice (Supplemental Figure 18, J–L) and aged WT mice in response to a CCL3 neutralization antibody (Supplemental Figure 18, M–O).

## Discussion

Aging alters the in vitro differentiation potential of BMSCs (27). We observed that BMSCs acquired a bias in the differentiation potential in favor of adipogenesis over osteogenesis. Although previous studies have demonstrated that osteoblast differentiation is negatively related to adipocyte differentiation (28), these results were contradicted by other studies in which no difference was observed and BMSCs from older donors exhibited decreased adipogenic differentiation potential compared to younger donors (29). The reasons for this discrepancy may be related to 2 factors: i) Due to the lack of conclusive markers and assays to identify BMSCs, it is possible that BMSCs isolated from different labs represent different subpopulations and therefore have different phenotypes, including differentiation potential. ii) The microenvironment undergoes significant changes when BMSCs are isolated from in vivo bone marrow and cultured in vitro, which may be sufficient to alter BMSC phenotypes. Most research on the relationship between BMSC age and differentiation have only assayed in vitro differentiation. Whether this occurs in vivo remains unclear.

Bone marrow is highly sensitive to age-related changes in intrinsic and extrinsic signals, including hormones, growth factors, adipokines, cytokines, transcription factors, and members of cellular signaling pathways (30). Previous studies have indicated that osteogenic and adipogenic differentiation are tightly linked, and it is believed that a bipotential osteoblast/adipocyte precursor exists, the commitment of which to either lineage results from the involvement of certain intrinsic and extrinsic signals, transcription factors, and cofactors (31). We believe that identifying the extrinsic signals in the age-related bone marrow microenvironment changes is significant to elucidate the molecular mechanisms underlying age-related skeletal diseases, such as senile osteoporosis. Therefore, in the current study, the levels of CCL family members in the peripheral blood serum were compared between young and aged mice. CCL3 accumulation was observed in the serum and bone marrow of aged mice compared with young mice; to the best of our knowledge, this is the first study to identify this phenomenon. More importantly, 5 lines of evidence were provided by the current study to support the involvement of CCL3 in the bone loss and bone marrow adiposity of aged mice: i) CCL3 levels in the serum and bone marrow were negatively associated with femur BV/TV and positively associated with BMAT femur volume. ii) Deletion of CCL3 increased in in vivo bone mass and decreased bone marrow adiposity of aged mice. iii) rCCL3 inhibited osteogenic differentiation potential and improved adipogenic differentiation potential of in vitro cultured BMSCs. iv) Deletion of CCL3 receptors, CCR1 and CCR5, from BMSCs abrogated the regulation of in vitro cultured BMSCs’ osteogenic/adipogenic differentiation by CCL3. v) In vivo blocking of CCL3 using a neutralization antibody ameliorated the trabecular bone loss and bone marrow adiposity of aged mice. These data highlight the role of CCL3 in skeletal aging and provide insights into the potential of CCL3 as a target to prevent, or treat, age-related bone diseases, such as senile osteoporosis.

The source of high-level CCL3 in the bone marrow of aged mice is an important question that needs to be answered; however, this is complicated by the highly complex microenvironment of bone marrow containing numerous cell types. CCL3 is typically expressed by stromal cells, HSCs (32), osteoclasts, and osteoblast precursor cells (33) under physiological conditions. Although isolation of all cell types in the bone marrow for comparison of CCL3 expression between young and aged mice would prove highly difficult, herein, HSCs, B cells, BMSCs, and BMAT were isolated and cultured in vitro. Our evaluation showed that BMAT exhibited the highest CCL3 secretion ability among these cell types. Considering the bone marrow adiposity of aged mice, we postulated that BMAT accumulation in the bone marrow of aged mice, at least in part, contributed to the high CCL3 expression. Our data are supported by those of a previous study, in which microarray of the adipocytes of obese and nonobese Pima Indians revealed that most differentially expressed inflammation-related genes were upregulated in the adipocytes, including CCL2, CCL3, CCL4, CXCL1, CXCL2, CXCL3, and CXCL12 (24). In the future, to determine which cell type is most important in regulating CCL3 expression in the bone marrow, conditional knockout mice should be generated using promoters expressed by specific types of stem cells (e.g., HSCs, B cells, BMSCs) and differentiated cells (e.g., adipocytes).

In the current study, when exogenous rCCL3 was used to treat BMSCs in vitro, young and aged BMSCs exhibited the same response: the promotion of adipogenic differentiation and the inhibition of osteogenic differentiation. However, the responses of young BMSCs to exogenous CCL3 were more significant than aged BMSCs. The reasons may be related to 2 factors: i) the basal levels of adipogenic and osteogenic differentiation potential of young and aged BMSCs — compared with young BMSCs, aged BMSCs have stronger adipogenic differentiation potential and weaker osteogenic differentiation potential — ii) more importantly, expression level of endogenous CCL3 from young and aged BMSCs. CCL3 expression was substantially upregulated by BMSCs of aged mice compared to young mice. In other words, high level of endogenous CCL3 expression could mask the effect of exogenous CCL3 on BMSCs to some extent.

CCL3 suppresses matrix mineralization and OCN production by upregulating ERK activity and downregulating OSX (26). Our study indicates that CCL3 inhibits the osteogenic differentiation potential of BMSCs by ERK-mediated DKK1 upregulation to inhibit the WNT/β-catenin signaling pathway. The MAPK signaling pathway is one of the most important modulators of osteogenic differentiation: the subtle control of ERK, p38, and JNK activity regulates the expression of several osteogenic transcription factors (34). In particular, the ERK pathway exerts profound effects according to the osteogenic differentiation stage. Continuous inhibition of ERK signaling promotes osteoblast development, whereas activation during late stages of osteogenic differentiation inhibits osteogenesis (34, 35). Our data suggest that exposing BMSCs to CCL3 activates ERK signaling, which in turn upregulates DKK1 expression to inactivate β-catenin activity.

The WNT pathway has been extensively studied in bone, where its dysregulation has been reported in the aging process (36). The canonical WNT pathway is indispensable in bone formation in humans, and is regulated by DKK1 (37), which antagonizes the WNT co-receptors LDL-receptor proteins 5 and 6. Given that DKK1 loss of function causes high bone mass, blocking these signals may serve as an effective strategy to ameliorate osteoporosis in elderly people, particularly postmenopausal women (38, 39). In the current study, the inhibition of calcium deposition and osteogenic differentiation marker genes’ expression by rCCL3 treatment was rescued in large part if not entirely by neutralization antibody–mediated DKK1 blockade, which highlights the important role of DKK1 in the regulation of BMSCs’ osteogenic differentiation by CCL3 and implies that other underlying molecular mechanisms are involved in the regulation of BMSCs’ osteogenic differentiation by CCL3 in addition to DKK1. In the future, further research will be required to explore whether CCL3 can regulate BMSCs’ osteogenic differentiation through other important osteogenic signaling pathways, including TGF-β/BMPs, Hedgehog, and Notch.

The most important findings of this study are related to the role of CCL3 in bone marrow adiposity and BMSCs’ adipogenic differentiation. In vivo deletion of *Ccl3* in knockout mice or via in vivo blocking with neutralization antibodies ameliorated bone marrow adiposity of aged mice, thus highlighting the role of CCL3 in bone marrow adiposity of aged mice. Moreover, C/EBPα was defined as the direct target of CCL3 and shown to favor BMSCs’ adipogenic differentiation. Our data revealed that CCL3 activated C/EBPα via STAT3 to promote BMSCs’ adipogenic differentiation. C/EBPα, which plays an essential role in the transcriptional activation of adipose differentiation (40), is highly expressed in rodent white and brown fat, and accumulates during adipocyte conversion with temporal kinetics in combination with acquisition of the differentiated phenotype (41).

Increased bone marrow fat is accompanied by age-dependent bone loss, as well as bone loss caused by estrogen deficiency and glucocorticoid excess (6, 42). Thiazolidinediones, activators of the key adipogenic transcription factor PPARγ, increase marrow adipocytes, decrease bone formation, and cause loss of trabecular and endocortical bone (43, 44). Further, the age-dependent increase in bone marrow adipocytes is associated with increased lipid oxidation (45) and increased PPARγ expression in marrow mesenchymal progenitors (46). These observations have formed the long-standing idea that increased marrow adipogenesis concomitantly decreases the generation of osteoblasts needed to refill resorption cavities created by osteoclasts during bone remodeling (47, 48), resulting in unbalanced remodeling and further leading to osteoporosis development (49). Nevertheless, whether excess adipocytes in the bone marrow lead to decreased bone mass and represent the cause of pathologic bone loss remains unclear, as conflicting results have been reported. For instance, mice with conditional PPARγ deletion in osteoblast progenitors show either no change or a small increase in femoral or spinal bone mass (50, 51). Similarly, bone mass is unaffected in mice lacking marrow adipocytes due to 11β-hydroxysteroid dehydrogenase deletion (52) or a loss-of-function mutation in the Kit receptor (53). Although the current study did not address this question, as we did not eliminate bone marrow adipocytes via genetic or pharmacologic methods in aged mice to observe the effects on bone mass, we demonstrated that the commitment of BMSCs to osteoblasts or adipocytes occurs in a mutually exclusive fashion. Moreover, we highlighted the role of CCL3 in aged mouse bone loss and bone marrow adiposity. For example, when CCL3 was ablated by genetic (knockout mice) or pharmacologic (neutralization antibody administration) methods, bone marrow adipocytes decreased and bone mass increased.

Notably, changes in the trabecular and cortical bone mass of aged mice were observed in response to treatment with a CCL3 neutralization antibody. Compared with in vivo *Ccl3* deletion in KO mice, in which both trabecular and cortical bone mass were enhanced, the CCL3 neu antibody only promoted trabecular bone mass without significantly affecting aged mice cortical bone mass. This discrepancy may have been caused by the duration of antibody administration; that is, 14-month-old mice were administrated CCL3 neu antibodies once every 10 days for 4 months. We speculate that with early administration of the antibody, CCL3 activity can be blocked more significantly.

**Figure 8:**
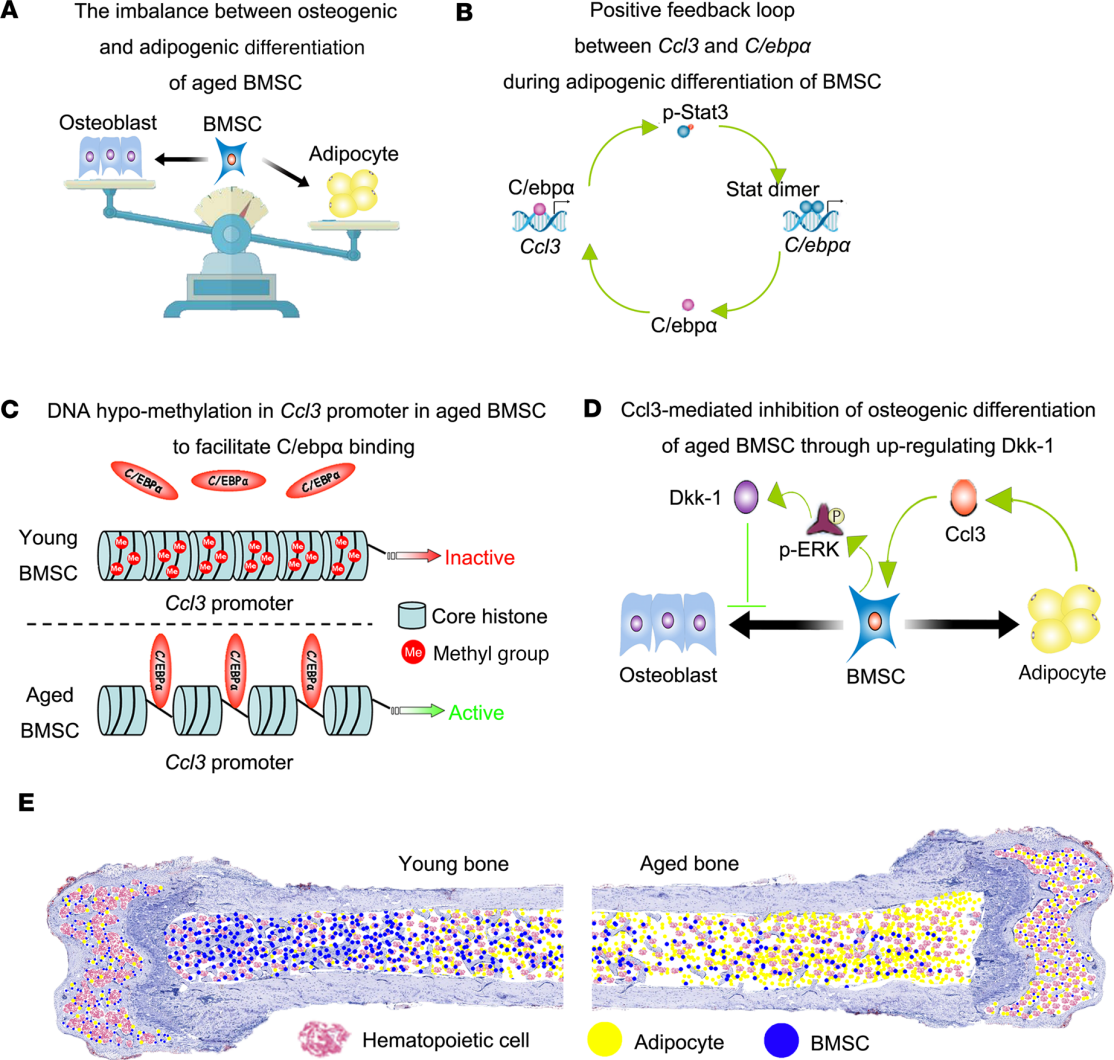
Potential role of CCL3 in bone loss and bone marrow adiposity of aged mice. (**A**) Imbalance between osteogenic and adipogenic differentiation of aged BMSCs. (**B**) Positive feedback loop between CCL3 and C/EBPα during adipogenic differentiation of BMSCs. (**C**) DNA hypomethylation of CCL3 promoter in aged BMSCs to facilitate C/EBPα binding. (**D**) Inhibition of osteogenic differentiation of aged BMSCs by CCL3 through ERK-mediated DKK1 upregulation. (**E**) Comparison of young and aged bone. Pink bubbles indicate hematopoietic cells, blue bubbles indicate BMSCs, and yellow bubbles indicate adipocytes in bone marrow.

In summary, this study demonstrates that CCL3 accumulates in the bone marrow of aged mice and triggers an imbalance between BMSC adipogenic and osteogenic differentiation (Figure 8A) by favoring adipogenesis via a positive feedback loop involving CCL3 and C/EBPα (Figure 8, B and C) and suppressing osteogenesis through upregulation of DKK1 activity (Figure 8D). As a result, bone loss and bone marrow adiposity occur (Figure 8E).

## Methods

### Animals.

C57BL/6 mice were purchased from SIPPR-BK Laboratory Animal Co. Ltd. *Ccl3*-KO mice (strain 002687) (10), *Ccr1*-KO mice (strain 032932) (54), and *Ccr5*-KO mice (strain 005427) (55) were purchased from The Jackson Laboratory. *Ccr1*-KO mice were crossed with *Ccr5*-KO mice to produce *Ccr1/5* DKO mice. Male mice were used in the current study. All mice were maintained under specific pathogen–free conditions in the animal facility of Shanghai Ninth People’s Hospital, Shanghai Jiao Tong University School of Medicine. Animals were euthanized via isoflurane inhalation anesthesia followed by cervical dislocation. Femurs were collected for micro-CT scanning, bone marrow cell preparation, and histomorphometry analysis. Peripheral blood serum was obtained by intracardiac puncture for antibody array analysis.

Fourteen-month-old mice were intraperitoneally administrated CCL3 neu antibody (4 mg/kg; MAB4502, R&D Systems, Bio-Techne) once every 10 days for 4 months. IgG (MAB005, R&D Systems, Bio-Techne) was used as a control.

### Micro-CT.

Femur bone mass and microstructure were evaluated by micro-CT as previously described (56). Femurs were collected and stored in 70% ethanol for further use. Bones were scanned by a micro-CT instrument (μCT-80, Scanco Medical AG). Standard nomenclature and guidelines for bone microstructure were recommended by the American Society for Bone and Mineral Research (57). Trabecular bone of the distal femur was evaluated in the secondary spongiosa, beginning about 0.6 mm proximal from the growth plate. The main parameters were BV/TV, Tb.N, Tb.Th, and Tb.Sp. The cortical region was measured at the femur midpoint with an isotropic pixel size of 21 μm and slice thickness of 21 μm; the average total cross-sectional area (mm^2^), bone area (mm^2^), and Ct.Th were calculated.

### Bone marrow adipocyte quantification.

Frozen sections of femur bone marrow cavity, fixed with paraformaldehyde and decalcified with EDTA, were stained with Oil Red O solution for 1 hour in a 60°C water bath. Parameters including Ad.Ar/Ma.Ar and Ad.N/Ma.Ar were quantified in a region within 50 μm distal from the proximal growth plate and 50 μm from the endocortical surface with a height 1.5 mm. Marrow area was calculated as total area – bone surface. Five sections of each staining were analyzed per animal. Bioquant software (Bioquant Image Analysis) was used to determine adipocyte number and area.

### Mechanical testing.

An Instron testing device was used in a 3-point bending test, which was performed at room temperature. The tests were performed on the femurs according to Jepsen et al. (58). A module contained 2 parallel supports with 10 mm spacing. The middle support could move in a vertical direction. Preloaded 0.5 N was applied to the mid-diaphysis to stabilize the bone. An increasing force with a displacement rate of 1 mm/min was applied until failure. The maximum load (N) and stiffness (N/mm) were calculated from the load displacement curve.

### Isolation of cell components in the bone marrow microenvironment of mice.

HSCs were isolated as previously described (56). MACS LS columns (Miltenyi Biotec) were used for either positive selection of c-Kit^+^ cells or negative deletion of lineage-negative (Lin^−^) cells. Cells were collected for further sorting. HSCs were gated for cell sorting by flow cytometry as follows: Lin^–^Sca1^+^c-Kit^hi^IL-7Rα^–^Flt3^–^Thy-1.2^+^.

B cells were isolated as previously described (25). Briefly, bone marrow cells were flushed from the bone marrow cavity and subchondral bone of femurs. Red blood cells (RBCs) from bone marrow were eliminated using RBC lysis buffer (Bio-Rad). B cells were then purified, based on CD19 expression using microbeads conjugated with anti-CD19 antibody (130-119-797, Miltenyi Biotec) and magnetic isolation (Miltenyi Biotec).

Based on a protocol described previously (59), BMAT was isolated from femurs by flushing the bone marrow and then high-speed spinning. The pellets containing the BMAT were then suspended in PBS to allow BMAT to float on the top of the liquid.

BMSCs were isolated as previously described (60). Femur bone marrow was suspended in PBS and passed through a 70 μm filter. The supernatants were stored for ELISA use. Filtered bone marrow cells were enriched for Lin^−^ cells using the RosetteSep system (Stemcell Technologies). The cells were incubated with a murine progenitor enrichment cocktail (19771, Stemcell Technologies) on ice for 30 minutes, washed, and then seeded into a flask at a density of 1 × 10^5^ cells/cm^2^ in α-MEM with 10% FBS. The media were changed after 3 days, and adherent cells were cultured with media changes twice each week. BMSCs (passage < 3) were used. For various purposes, BMSCs were treated in vitro with neu CCL3 antibody (0.5 μg/mL; R&D Systems, Bio-Techne), recombinant mouse CCL3 protein (5 ng/mL; R&D Systems, Bio-Techne), AG490 (50 μM; MilliporeSigma), 5′-aza (10 μM; MilliporeSigma), or DKK1 neutralization antibody (10 μg/mL; AF1096, R&D Systems, Bio-Techne).

### Osteogenic and adipogenic differentiation of BMSCs.

For osteogenic differentiation, BMSCs were treated with 100 nM dexamethasone (Dex), 10 mM β-glycerophosphate disodium, and 50 μg/mL ascorbic acid (all from MilliporeSigma).

For adipogenic differentiation, confluent BMSCs were treated with complete adipogenesis induction medium including 10 μg/mL of insulin, 0.5 mM methylisobutylxanthine, and 1 μM Dex (all from MilliporeSigma). The point of induction was designated as day 0. On day 3, cells were treated with 10 μg/mL insulin. On day 6, complete adipogenesis induction medium was again added. The whole adipogenesis induction process lasted 5 cycles (~30 days).

### Antibody array.

As previously described (61), absolute quantitative sandwich-based antibody array (RayBio) was used to detect 14 CCL family members. The detection antibodies were biotin-labeled and mixed as a cocktail reagent for later use. After blocking, the antibodies were incubated with peripheral blood serum and subsequently washed. The biotinylated detection antibody cocktail was added to the arrays. Following incubation and washing, the array slides were incubated with a streptavidin-conjugated fluor (HiLyte Fluor 532, from Anaspec). Fluorescent signals were visualized using a laser-based scanner system (GenePix 4200A, Molecular Dynamics).

### ChIP.

Cells were fixed with 1% formaldehyde for 10 minutes at 37°C. The crude nuclei were subjected to sonication to produce chromatin fragments of about 500 bp length. The antibodies included anti-C/EBPα (catalog PA5-77911, Thermo Fisher Scientific) and anti–p-STAT3 (catalog 9145, Cell Signaling Technology). Isotype IgG (catalog 61656, Cell Signaling Technology) was used as a negative control. Primary antibodies (2–5 μg) and samples were incubated overnight at 4°C with gentle shaking. Primer sequences for ChIP-qPCR are listed in Supplemental Table 2. ChIP regions within the CCL3 and C/EBPα promoter are displayed in Supplemental Figures 5 and 7.

### Bisulfite sequencing PCR.

Genomic DNA was denatured for 15 minutes at 50°C with 2 M NaOH. Low-melting agarose beads (2 %, v/v) were added to the DNA solution, and agarose beads were produced by adding 10 μL of the DNA/agarose mixture into cold mineral oil. The beads were then incubated with freshly prepared hydroxyquinone (10 mM, MilliporeSigma) and sodium bisulfite (40.5%, w/v, MilliporeSigma) at 50°C for 16 hours. Reaction was terminated by adding NaOH (0.3 M) for 10 minutes at room temperature. The primer sequences for PCR are listed in Supplemental Table 3. The amplified PCR products were subcloned into the pMD19-T vector (Takara). Twelve clones were sequenced per sample, and the sense strands were used to evaluate CpG site methylation.

### Data availability statement.

All data that support the findings of this study are openly available in Mendeley at http://dx.doi.org/10.17632/ht5846dgdk.1 Additional methods details may be found in Supplemental Methods.

### Statistics.

Student’s 2-tailed *t* test was used for 2-sample comparisons. One- and 2-way ANOVAs with Tukey’s post hoc test were used for multiple comparisons. *P* < 0.05 was considered statistically significant. All data are presented as means ± SD unless otherwise specified.

### Study approval.

All experiments were performed with the protocol approved by the Animal Care and Use Committee of Shanghai Ninth People’s Hospital, Shanghai Jiao Tong University School of Medicine (HKDL2018305).

## Author contributions

QF, DY, GL, and ZZ conceived and designed the research. ZZ, SZ, CM, SH, LX, JL, GL, DY, and QF performed the experiments. ZZ, SZ, HL, CM, GL, DY, and QF analyzed the data. QF, DY, and ZZ wrote the manuscript.

## Supplementary Material

Supplemental data

## Version 1 11/15/2022

In-Press Preview

## Version 2 01/10/2023

Electronic publication
