# Supplementary material for: CCL3 in the bone marrow microenvironment causes bone loss and bone marrow adiposity in aged mice
Source: JCI Insight. 2023 Jan 10;8(1):e159107. doi: 10.1172/jci.insight.159107 (PMC9870077; doi:10.1172/jci.insight.159107)
Supplement: Supplemental data [file jciinsight-8-159107-s009.pdf]

Supplementary Materials and Methods

Bone histomorphometry

For dynamic bone histomorphometry, mice were subcutaneously injected with calcein (15 mg/kg, Sigma, St. Louis, MO, USA) on day 10 and day 3 before euthanasia. Following fixation and embedding, femurs were sectioned (50 μm) and double calcein labeling was imaged. BFR was calculated as inter-label width/labeling period. Five sections of each staining were analyzed per animal. The sampling site for the distal femoral metaphysis was located 0.25–1.25 mm proximal to the growth plate and 0.1 mm from cortical bone. Images were analyzed using ImageJ in a blinded manner.

For static histomorphometry, femurs were decalcified, embedded in paraffin, and sectioned in 5 μm onto slides. Hematoxylin and eosin (H&E) stain was used to measure bone formation parameters including Ob.S/BS and Ob.N/B.Pm. Tartrate resistant acid phosphatase (TRAP) stain was used to measure bone resorption parameters including Oc.S/BS and Oc.N/B.Pm. Five sections of each staining were analyzed per animal. The sampling site for the distal femoral metaphysis was located 0.25-1.25mm proximal to the growth plate and 0.1mm from cortical bone. Images were analyzed using ImageJ in a blinded manner.

ALP, AR, and Oil red O staining

For ALP staining, cells were fixed with 4% paraformaldehyde at 4°C and then incubated in 0.1% naphthol AS-MX phosphate (Sigma) and 0.1% fast red violet LB salt (Sigma) in 2-amino-2-methyl-1,3-propanediol (Sigma) for 10 min at room temperature.

For AR staining and quantification, cells were fixed with 4% paraformaldehyde at 4°C and then incubated in 40 mM AR solution (pH 4.2) for 30 min at 37°C. After imaging, decalcification was performed in 0.1 M HCl for 8 h at 4 °C. Then 10 μl of samples were mixed with 0.5 mL of methyl thymol blue and 0.5 mL of alkaline solution. Absorbance was quantified at 610 nm.

For Oil red O staining, cells were fixed with 4 % paraformaldehyde and stained

in Oil Red O solution for 1 h in a 60 °C water bath.

qRT-PCR

Total RNA was prepared using the RNeasy Mini Kit (Qiagen, Valencia, CA, USA). Single-stranded cDNA was reverse transcribed from 1 μg total RNA using oligo-dT primers. Quantitative PCR was performed on an ABI Prism 7500 system (Applied BioSystems, Foster City, CA, USA) employing SYBR Green PCR Master Mix (Takara Bio Inc., Otsu, Japan). Cycling conditions was as follows 94 °C, 5 s; 60 °C, 34 s; and 72 °C, 40 s for 40 cycles. Actb (β-actin) and Hprt were used as internal controls. Primer sequences were listed in Table S1.

Western blot

Total protein was prepared by lysing cells on ice for 30 min in a buffer (50 mM Tris-HCl, 150 mM NaCl, 1% Nonidet P-40, and 0.1% SDS supplemented with protease inhibitors). The proteins were separated by SDS-PAGE, transferred to a PVDF membrane, and detected using anti-CCL3 (AF450, R&D systems), anti-p-STAT3 (Y705; 9145, Cell Signaling Technology, Danvers, MA, USA), anti-STAT3 (9139, Cell Signaling Technology), anti-DKK-1 (MAB1765, R&D systems), anti-active β-catenin (19807, Cell Signaling Technology), anti-p-ERK (4370, Cell Signaling Technology), anti-ERK (4695, Cell Signaling Technology), anti-HPRT (ab10479, Abcam), and anti-GAPDH (2118, Cell Signaling Technology) antibodies.

Luciferase reporter assay

Cells were cultured in 24-well plates. All plasmids were prepared using QIAGEN plasmid purification kit. Transient transfection was performed using Lipofectamin 3000 (Invitrogen) and phRL-SV40 vector (Promega, Madison, WI, USA) was used as transfection efficiency control. Forty-eight hours after transfection, the cells were lysed and both firefly and renilla luciferase activities were evaluated using Dual-luciferase reporter assay system (Promega).

In vitro methylation assay

M.SssI (New England Biolabs, Ipswich, MA, USA) (2 U/μg DNA) was

employed to methylate CpG sites in vitro for 6 h at 37 °C and then inactivated for 15 min at 65 °C. Methylated DNA fragments and vectors were ligated and purified by phenol/chloroform extraction and ethanol precipitation.

Osteoclast differentiation assay

Animals were euthanized using isoflurane inhalation anesthesia followed by cervical dislocation. The femur and tibiae of mice were washed with serum-free α-MEM. Bone marrow cells were incubated with α-MEM supplemented with 10% FBS, penicillin (100 U/ml) and streptomycin (100 mg/ml) for 24 h. Non-adherent cells were harvested, and induced to bone marrow monocytes (BMMs) using M-CSF (20 ng/ml). Cells were cultured in the humidified atmosphere at 37 °C and 5% CO2.

BMMs were incubated in 48-well plate in α-MEM containing M-CSF (20 ng/ml) plus RANKL (100 ng/ml) to form mature osteoclasts. Osteoclasts differentiation was observed by Tartrate resistant acid phosphatase (TRAP) staining. TRAP+ cells with more than three nuclei were considered as mature osteoclasts. TRAP+ cells with more than five nuclei were considered as large osteoclasts.

Immunohistochemistry

Femurs were fixed in 4% paraformaldehyde, decalcified, and embedded in paraffin. Serial sections were cut at 5 μm thickness. Slides were incubated with primary antibody against CCL3 (AF450, R&D systems), DKK-1 (MAB1765, R&D systems) and active β-catenin (19807, Cell Signaling Technology) overnight at 4 °C. For immunohistochemical staining, a horseradish peroxidase-streptavidin detection system (Dako) was used to detect immuno-activity. Isotypoe IgG was used as s negative control (Fig. S19). Five slides in each sample were selected to enumerate the stained cells per ×100 optical fields under a light microscope.

Supplementary tables

Table S1: Primer sequences used in quantitative RT-PCR

Gene	Primer sequences (5'-3')	Product length (bp)
Ccl3	TACAAGCAGCAGCGAGTACC (F)	246
	GAGCAAAGGCTGCTGGTTTC (R)	
Runx2	TTCAACGATCTGAGATTTGTGGG (F)	221
	GGATGAGGAATGCGCCCTA (R)	
Opn	CCAGCAGCTCACACTGAAGA (F)	207
	AAAAGTCTGTCGGAGTGCTGA (R)	
Bsp	ATGGAGACGGCGATAGTTCC (F)	148
	CTAGCTGTTACACCCGAGAGT (R)	
Ocn	GCTACCTTGGAGCCTCAGTC (F)	71
	AGGGTTAAGCTCACACTGCT (R)	
Ppar-γ	TTCGCTGATGCACTGCCTAT (F)	224
	GCAACCATTGGGTCAGCTCT (R)	
C/ebpα	TTCGGGTCGCTGGATCTCTA (F)	164
	TCAAGGAGAAACCACCACGG (R)	
aP2	TTTGGTCACCATCCGGTCAG (F)	200
	CCAGCTTGTCACCATCTCGT (R)	
Glut4	CTTGGCTCCCTTCAGTTTG (F)	130
	TGCCTTGTGGGATGGAAT (R)	
Dkk-1	TCTCTATGAGGGCGGGAACA (F)	156
	TTTCGGCAAGCCAGACAGAT (R)	
NFAT2	GAGAGGTCGGACTCGGGA (F)	239
	ACACATAACTGTAGTGTTCTTCCT (R)	
CATK	CTGCGGCATTACCAACATGG (F)	192
	ACTGGAAGCACCAACGAGAG (R)	
CTR	CGGCAGGCACTGCTAAGGA (F)	222
	ACTTACCTTAGGTGGATGCAGG (R)	
β-actin	GGCTGTATTCCCCTCCATCG (F)	154
	CCAGTTGGTAACAATGCCATGT (R)	
Hprt	GGTTAAGCAGTACAGCCCCA (F)	224
	ACTTGCGCTCATCTTAGGCT (R)	

F: forward, R: reverse

Table S2: Primer sequences used in ChIP-qPCR

Gene	Primer sequences (5'-3')	Product length (bp)
Ccl3	CACAGGCCCATCTCACAAGT (F)	297
	CAAAACCTTTCACTATGAAA (R)	
C/ebpα	AGCCAGGTCCACCAGGCTCC (F)	276
	AAAAGGCTGTGGCGGAGAGCC (R)	

F: forward, R: reverse

Table S3: Primer sequences used in bisulfite sequencing PCR

Name	Primer sequences (5'-3')	Product length (bp)
BS-PCR region1	AATGGAAAAAATATTTTTAGG (F)	305
	AATAAACACTCCAAACCCATT (R)	
BS-PCR region2	AATGGGTTTGGAGTGTTTATT (F)	390
	CTTATCAACAAAACAAAAAA (R)	

F: forward, R: reverse

Supplementary figures

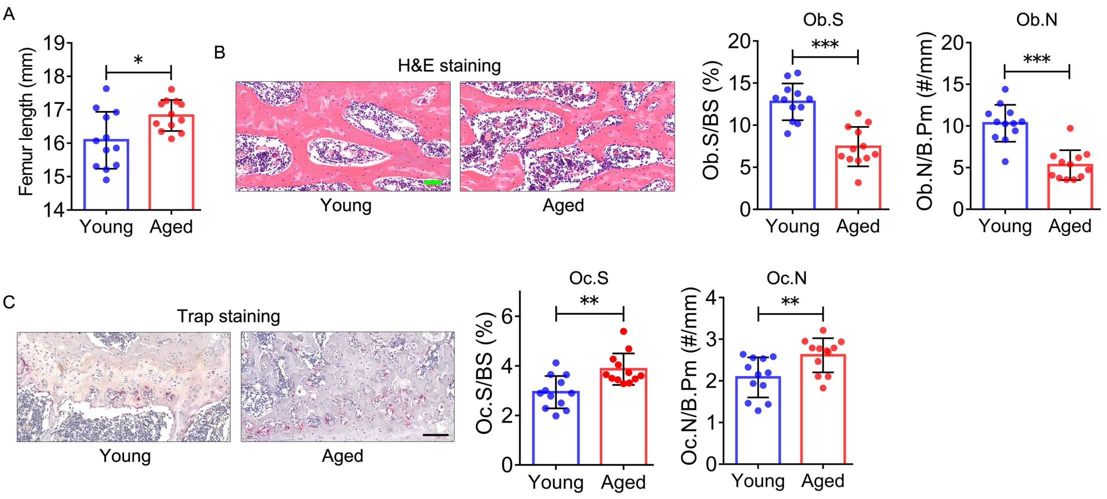

Figure S1

Figure S1: Static bone histomorphometry of young and aged mice. (A) Femur length of young and aged mice (n=12). **(B)** H&E staining and the histomorphometric analysis of osteoblast surface (Ob.S/BS) and number (Ob.N/B.Pm) in femur of young and aged mice (n=12). Scale bar: 50 μm. **(C)** TRAP staining and the histomorphometric analysis of osteoclast surface (Oc.S/BS) and number (Oc.N/B.Pm) in femur of young and aged mice (n=12). Scale bar: 100 μm. All the data were obtained from three independent experiments. The images and numerical data are representative. Data were shown as the means ± s.d. *: $p<0.05$, **: $p<0.01$, ***: $p<0.001$.

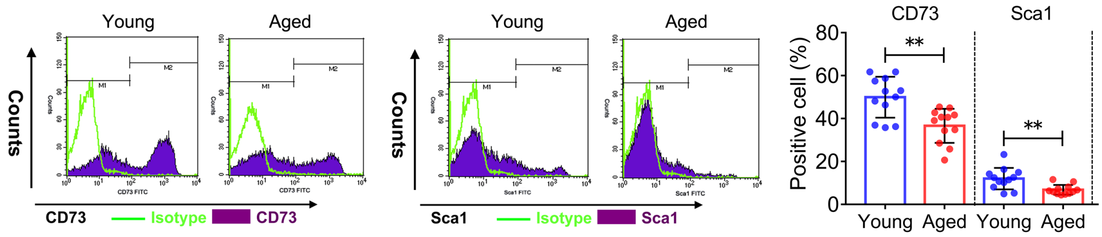

Figure S2

Figure S2: BMSCs number (CD73^{+} and Sca1^{+} cells) in bone marrow of femur of young and aged mice determined by flow cytometry (n=12). **: p<0.01.

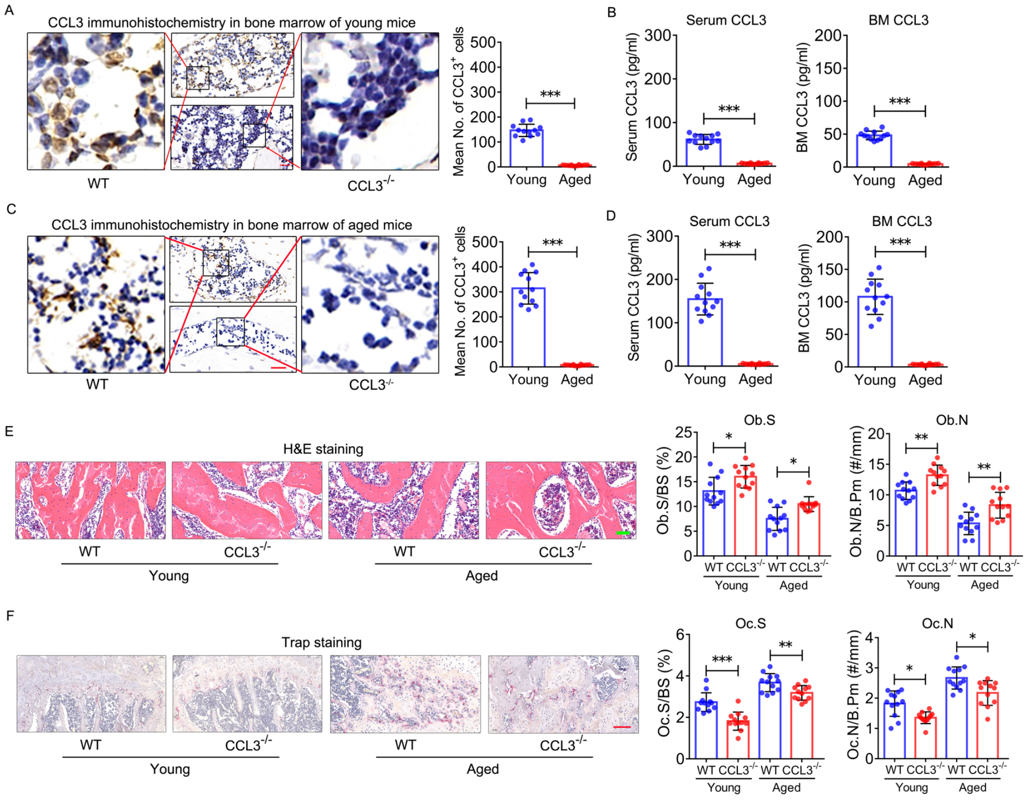

Figure S3

Figure S3: CCL3 expression in the bone marrow and serum and static bone histomorphometry of CCL3$^{-/-}$ mice. (A) CCL3 expression in femur bone marrow of young WT and CCL3$^{-/-}$ mice determined by immunohistochemistry and the quantification of CCL3+ cells. Scale bar: 40 μm. **(B)** CCL3 levels in the peripheral blood serum and femur bone marrow of young WT and CCL3$^{-/-}$ mice determined by ELISA (n=12). **(C)** CCL3 expression in femur bone marrow of aged WT and CCL3$^{-/-}$ mice determined by immunohistochemistry and the quantification of CCL3+ cells. Scale bar: 40 μm. **(D)** CCL3 levels in the peripheral blood serum and femur bone marrow of young WT and CCL3$^{-/-}$ mice determined by ELISA (n=12). **(E)** H&E staining and the histomorphometric analysis of Ob.S/BS and Ob.N/B.Pm in femur of WT and CCL3$^{-/-}$ mice (n=12). Scale bar: 50 μm. **(F)** TRAP staining and the quantification of Oc.S/BS and Oc.N/B.Pm of femur of WT and CCL3$^{-/-}$ mice. Scale bar: 100 μm. All the data were obtained from three independent experiments. The images and numerical data are representative. Data were shown as the means ± s.d. *: $p<0.05$, **: $p<0.01$, ***: $p<0.001$.

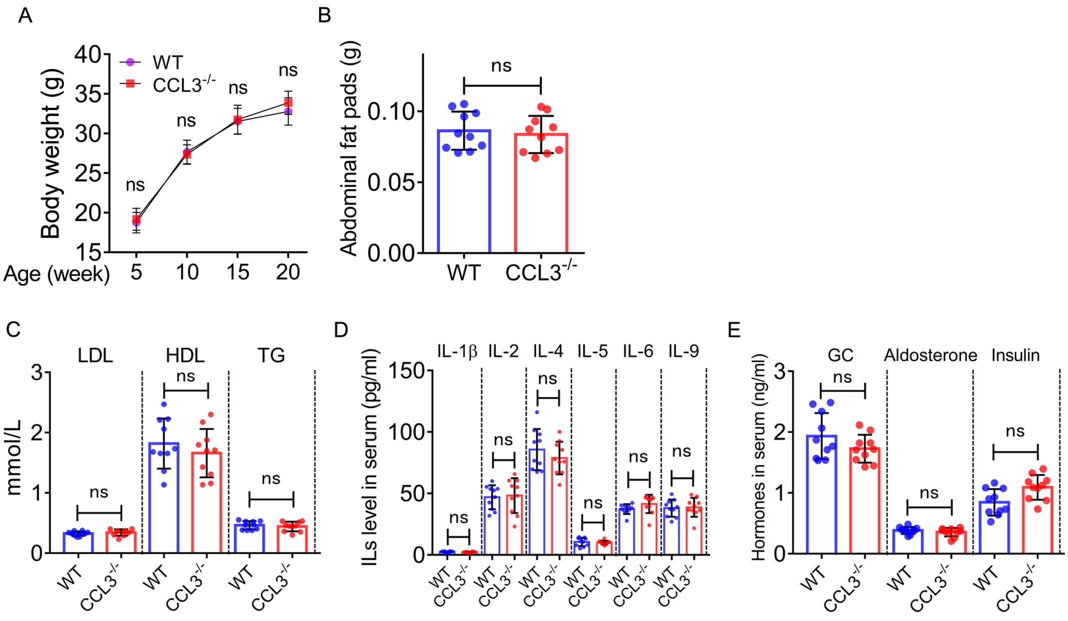

Figure S4

Figure S4: Phenotypes of CCL3$^{-/-}$ mice. (A) Body weight of WT and CCL3$^{-/-}$ mice at 5-, 10-, 15-, and 20-week age (n=10). **(B)** Abdominal fat pads weight of WT and CCL3$^{-/-}$ mice (n=10). **(C)** Serum levels of lipid metabolism markers including low density lipoprotein (LDL), high density lipoprotein (HDL), and triglyceride (TG) of WT and CCL3$^{-/-}$ mice (n=10). **(D)** Serum levels of inflammatory factors including interleukin-1β, 2, 4, 5, 6, and 9 (n=10). **(E)** Serum levels of endocrine function markers including glucocorticoid (GC), aldosterone, and insulin (n=10). All the data were obtained from three independent experiments. The images and numerical data are representative. Data were shown as the means ± s.d. ns: no significance.

C/EBPα promoter sequence

-1249bp

GCGGCCGCGATCCAGGTGGAGGGTGAACGAGACGCCACGTGGCTGGGAGCCG
ATGCACGCAAACTCCTACCCACAGCCGCGAGCCTGTAGGCGGCGCGGCGCGG
AGGGCTCCCAAGTGGGTGCTCGAAAGGCTTCGTAGCTAGGAATTGGACACCCG
AGCTACCGAGATTAGTGCCCCCATGAATGACAGTAGGGAAAGAAAACTGTGTCTT
CAGGCCCCTGGCTATGGGCCCCGCCTGGGGATCACAGTCCCCGATTCAAGTTC
ACTCCTCTCCAACACCCTGCCCTCTCGCGGCCCCTGTGCGCTCTCCTTAGGGTC
CTTTTCCGCGAGGCTCAGAGGACCCACCGGCTGGTCCCGGGCGTGGGGTGGT
GGTGTCCCGAACACTTGACTAGAGTGCTCCACGCTGGGTAGCAACGTCTGCCT
GGTAAGCCTAGCAATCCTATCGCTCTGGCCTGGAGACGCAATGAAAAAGAAAGT
TTTCCAGCCTAGGCGAGTGGACGAGCCAGGTCCACCAGGCTCCGGGGTTAGCG
GCCGCCTCCGCTCCCCGCCGGGTCCTAGCGCCCTACTACTCTGAAGAGCCCGT
GGGACCCTGTAGTTCTAGAGAAGCTGGGCGAAAGAGAGGTGCTCTGCCTGGAA
GCCGTGGGGTCGCGTGGAGTTCAGAGAAAAAGACGCACAATCTCTGCGCTCCC
GGCCTCGCCACTCGGCGGTGCGCGCTAGGTTGCTGGTCCAAAGCAGTCTCCAA
CCTCCCGCGCCGCGGCTCTCCGCCACAGCCTTTTAGAAATCCGGGTGGAGAC
AGGCCTAGTCCCAGCTTTTAACACAAGTCTGCACTACGGTAGCTCAAAACCAACA
TTCTCTCTCCAAACGCTCCCCAACCTCCACCTCCCCTCGCTCGGCCTCTAGATG
CTCCCGGGCTCCCTAGTGTTGGCTGGAAGTGGGTGACTTAGAGGCTTAAAGGA
GGGGCGCCTAACCACGGACCACGTGTGTGCGGGGGCGACAGCGCCGCCGGG
GTGGGGCTGAGCGCTGCAAGCCGGGTTCGCCTTGCAGCGCAGGAGTCAGTGG
GCGTTGCGCCACGATCTCTCTCCACTAGCACTATGCTCCCCACTCACCGCCTTG
GAAAGTCACAGGAGAAGGCGGGCTCTAAGACCCAGCAGGCACCATCCTACTGG
CGCCTTCGATCCGAGACCCGTTTGGACACCAGGGGGCGATGCCCGACCCTCTA
TAAAAGCGGTCCCCGCGCGGGCCTGGCCATTCGCGACCCGAAGCT

+17bp

Transcription start site Putative STAT3 binding site ChIP region

Figure S5

Figure S5. C/EBPα promoter sequence investigated in the current study. The DNA sequence of -1249bp~+17bp around C/EBPα transcription start site was shown. Transcription start site was labeled in red, putative STAT3 binding site was labeled in blue and ChIP region was labeled in green.

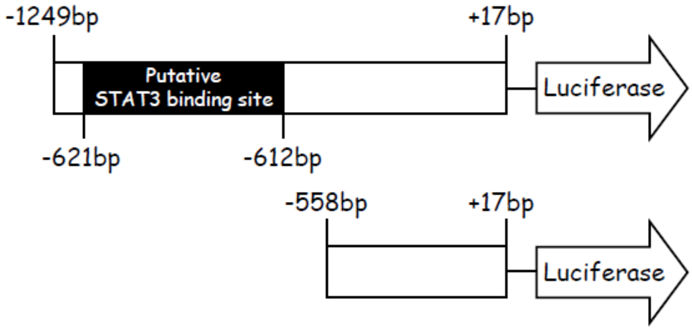

Figure S6

Figure S6: Diagram of C/EBPα promoter-driven luciferase reporter gene vectors. Putative STAT3 binding site is -621bp to -612bp.

Ccl3 promoter sequence

-1016bp

CCACTCACAGGCCCATCTCACAAGTTTTAATAATTCTTTAAAGTTTTTATTTT
TAGTGATTTTTTAAAAAAAAATTCCAGTATTATTTATTTATAATGCCTGGGTTT
GGTGTGAAAAACTGTATATTTCCAGATATTTGTAGAATTCTTCACTTTGTAA
AACTTGTACAATGTTTCTTGATATTTTTATCACCAATGTTATGAGTGTACTTA
TGTCATTTTAATTTTAAGAGCTTCATAAACAAGCAATCCAATGTATATACTAT
AATTTAATTTATAATTTTTTCATAGTGAAAGGTTTTGTTTCTTTAGTTTCAAGT
TATAATGACAATGGACCAGCTGTCTCAATATTTAGAGATGGCTTTACATTTG
GGTTGTTTCCCCAAAAATCTTTCCTAGAAAGGCTATTTCTAGCTTAATGGA
AAAAATATTTTTAGGCTTCTCACATATTAACGCCCACACTTGAAGTATCTTTA
TGATCCACAACTAATGTTCAACCCAGTGGCCTAGTCACTTTGCGGCTGAT
GATTGGACAAATGTCTTAAATGTGACCGCGTCTTCCCCATTGCTCGAGCT
GGAGATGCTTTTCCTCAGTCCCTCACTGTGGTGGTAGGAGGGGCTGTTG
TGCCACCATCCACAGCTATTCTTCCATGTGGTTAGAGAAGCTGAGAAATAA
CGAGAGTTTCCGTTCCATGGCAAGGAATGGGTTTGGAGTGTTTATTCACA
GAACGGTTCTCATGAGATGGGACCAGCTAAGAATAGCCCTGGGTTGACA
CTGTCCTACCTCCTCCTGCTCATAAGAGAAACTACTTCCCCACAAGAAGA
AAGAATAGGTCACGAGTTGAGAGCTGAGACTTATATCTCAGAGATGCTATT
CTTAGATATCCTGGGCCCCTGTGGTCACTGTGGACCCTGGGTTGTGTAAT
ATCCATCATGACACCATTGCTGTGCTTAAAATTTTCCCTCCTCAGCCCCGG
ATTCCATTTCCTCATCTGCTAGGGCTACCTATAAGAGAAGGGGCATATGGC
TTCAGACACCAGAAGGATACAAGCAGCAGCGAGTACCAGTCCCTTTTCTG
TTCTGCTGACAAGC

+75bp

Transcription start site

Putative C/EBPα binding site

ChIP region

CG sequence

Figure S7

Figure S7. Ccl3 promoter sequence investigated in the current study. The DNA sequence of -1016bp~+75bp around Ccl3 transcription start site was shown. Transcription start site was labeled in red, putative C/EBPα binding site was labeled in blue, ChIP region was labeled in green and CG sequences were labeled in yellow.

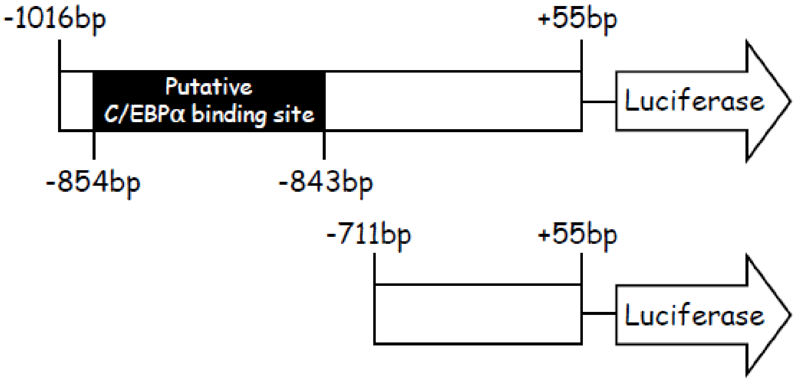

Figure S8

Figure S8: Diagram of Ccl3 promoter-driven luciferase reporter gene vectors. Putative C/ebpα binding site is -854bp to -843bp.

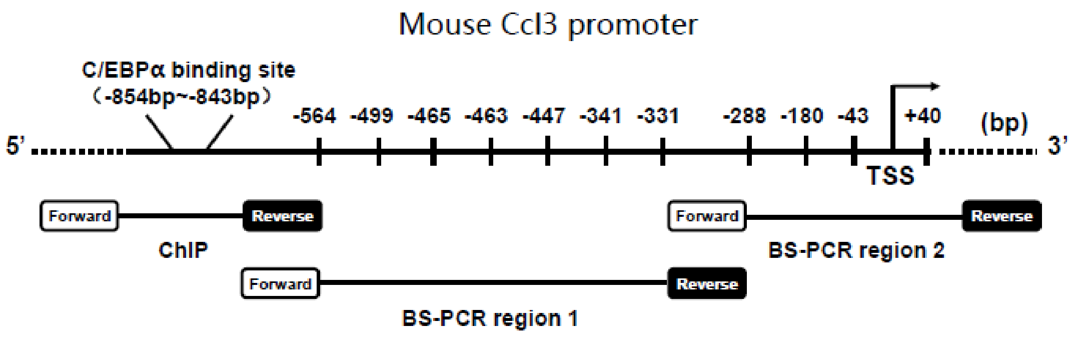

|: CG sequence; TSS: Transcription start site; BS-PCR: Bisulfite Sequencing PCR

Figure S9: Diagram of mouse Ccl3 promoter. Analysis of DNA methylation and transcription factor binding sites in Ccl3 promoter revealed 11 CpG sites and a putative C/EBPα binding site around the transcription start site.

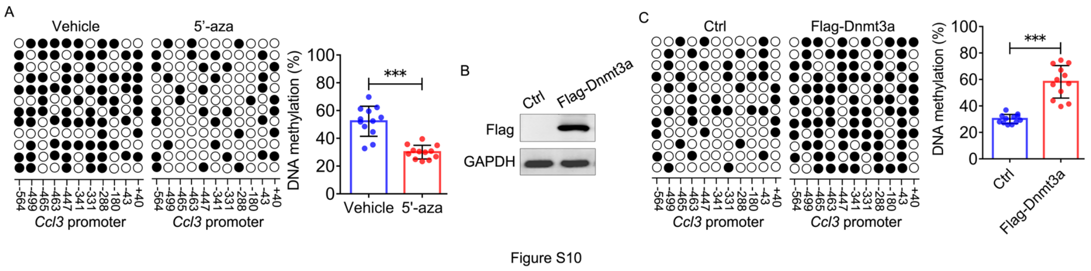

Figure S10: DNA methylation status of 11 CG sites within proximal CCL3 promoter in young BMSCs in response to 5'-aza treatment and in aged BMSCs in response to Dnmt3a overexpression. (A) DNA methylation percentage of 11 CG sites determined by bisulfite sequencing PCR within proximal CCL3 promoter in young BMSCs in response to 5'-aza treatment (n=12). **(B)** Lentivirus-mediated stable overexpression of Dnmt3a with Flag tag determined by western blot in aged BMSCs. GAPDH was used as loading control. **(C)** DNA methylation percentage of 11 CG sites determined by bisulfite sequencing PCR within proximal CCL3 promoter in aged BMSCs in response to Dnmt3a overexpression (n=12). All the data were obtained from three independent experiments. The images and numerical data are representative. Data were shown as the means ± s.d. ***: $p<0.001$.

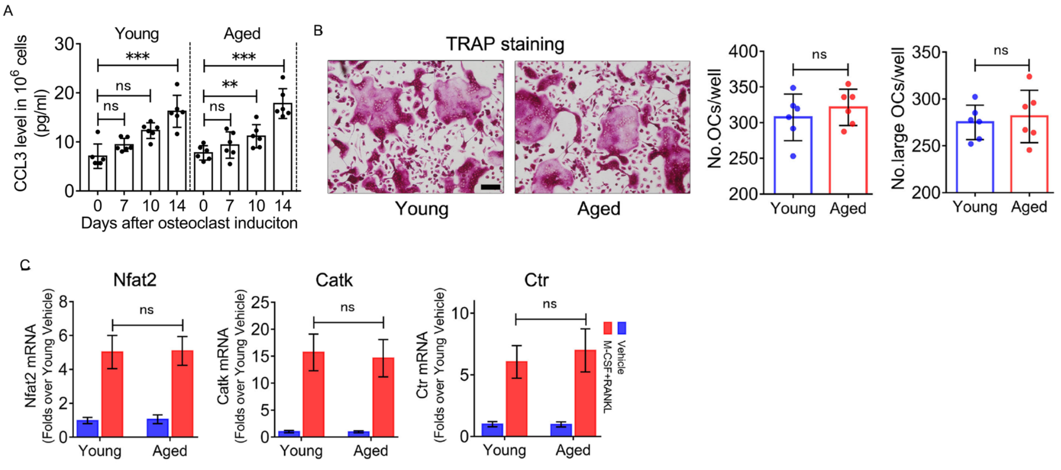

Figure S11

Figure S11: Osteoclast differentiation and CCL3 secretion from BMMs of young and aged mice. (A) CCL3 secretion determined by ELISA when BMMs from young and aged mice was induced to undergo osteoclastic differentiation (n=6). **(B)** TRAP staining images and quantificaiton of *in vitro* BMMs culture from young and aged mice in response to M-CSF and RANKL treatment. Scale bar: 200 μm. **(C)** mRNA expression of osteoclast differentiation markers including NFAT2, CATK and CTR determined by qRT-PCR when BMMs from young and aged mice was induced to undergo osteoclastic differentiation (n=6). All the data were obtained from three independent experiments. Data were shown as the means ± s.d. *: p<0.05, **: p<0.01, ***: p<0.001, ns: no significance.

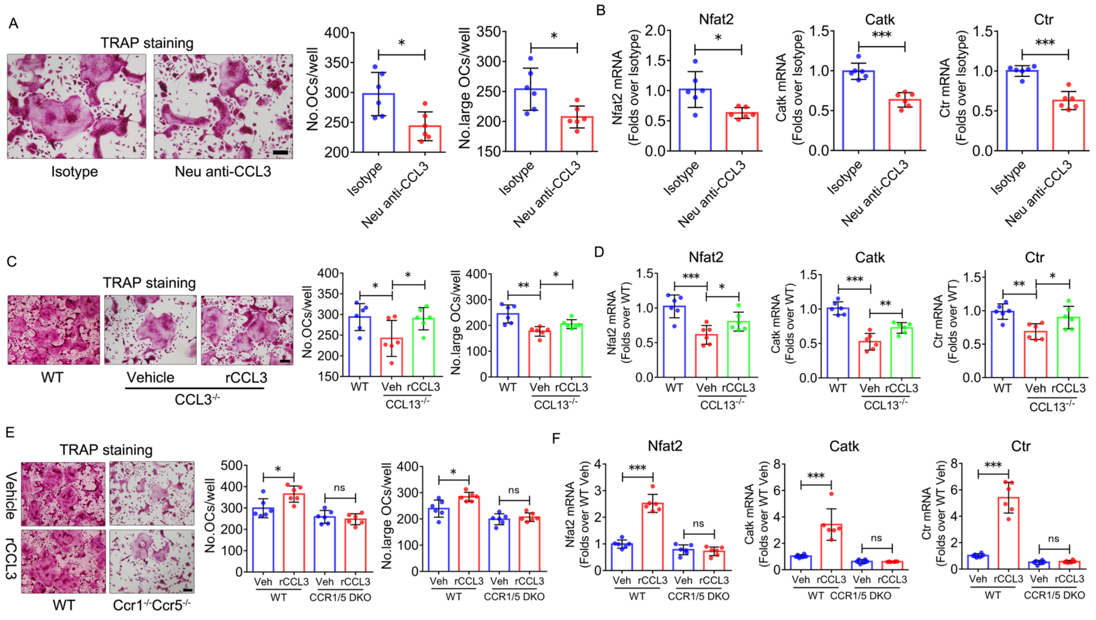

Figure S12

Figure S12: Role of CCL3 in osteoclast differentiation of BMMs. (A) TRAP staining images and quantificaiton when BMMs from young mice was induced to undergo osteoclastic differentiation in combination with neutralization antibody against CCL3 treatment. Scale bar: 200 μm. **(B)** mRNA expression of osteoclast differentiation markers including NFAT2, CATK and CTR determined by qRT-PCR when BMMs from young mice was induced to undergo osteoclastic differentiation in combination with neutralization antibody against CCL3 (n=6). **(C)** TRAP staining images and quantificaiton when BMMs from CCL3$^{-/-}$ and WT mice was induced to undergo osteoclastic differentiation in combination with recombinant CCL3 treatment. Scale bar: 200 μm. **(D)** mRNA expression of osteoclast differentiation markers including NFAT2, CATK and CTR determined by qRT-PCR when BMMs from CCL3$^{-/-}$ and WT mice was induced to undergo osteoclastic differentiation in combination with recombinant CCL3 (n=6). **(E)** TRAP staining images and quantification when BMMs from CCR1$^{-/-}$CCR5$^{-/-}$ and WT mice was induced to undergo osteoclastic differentiation in combination with recombinant CCL3 treatment. Scale bar: 200 μm. **(F)** mRNA expression of osteoclast differentiation markers including NFAT2, CATK and CTR determined by qRT-PCR when BMMs from CCR1$^{-/-}$CCR5$^{-/-}$ and WT mice was induced to undergo osteoclastic

differentiation in combination with recombinant CCL3 (n=6). All the data were obtained from three independent experiments. Data were shown as the means ± s.d. *: $p<0.05$, **: $p<0.01$, ***: $p<0.001$, ns: no significance.

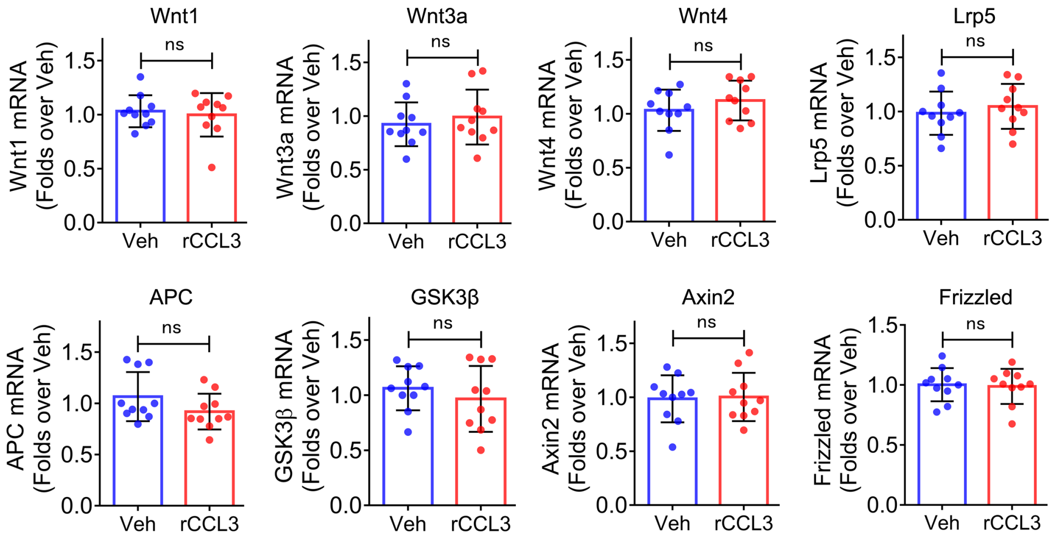

Figure S13

Figure S13: mRNA expression of Wnt/β-catenin signaling pathway members including Wnt1, Wnt3a, Wnt4, Lrp5, APC, GSK3β, Axin2, and Frizzled, determined by qRT-PCR, in BMSCs in response to rCCL3 treatment (n=10). GAPDH was used as internal control. All the data were obtained from three independent experiments. Data were shown as the means ± s.d. ns: no significance.

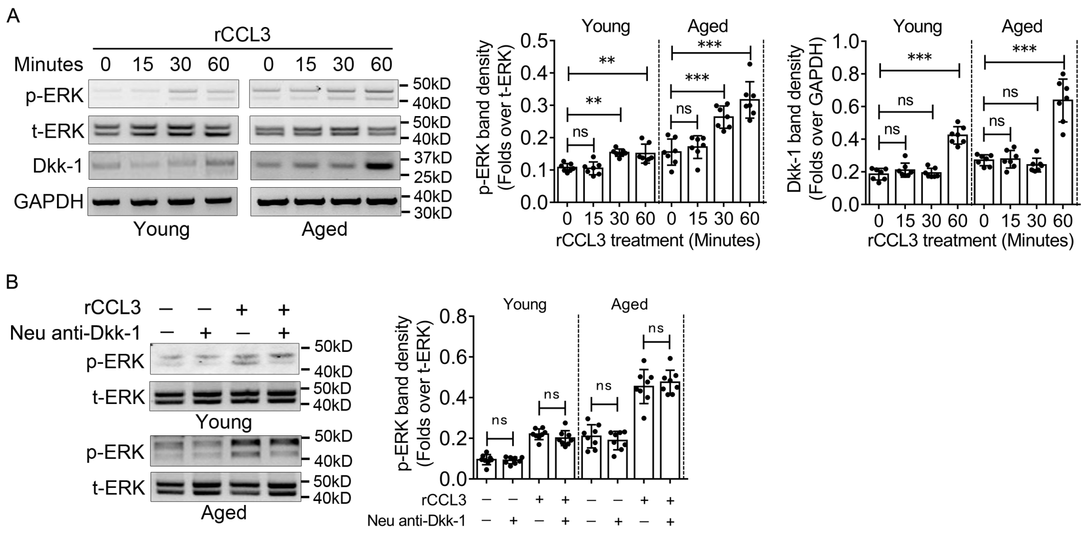

Figure S14

Figure S14: The phosphorylation of ERK in young and aged BMSCs in response to rCCL3 and/or neutralization antibody against Dkk-1 treatment. (A) The phosphorylation of ERK and Dkk-1 expression, determined by western blot, in young and aged BMSCs in response to 15-, 30-, and 60-minute rCCL3 treatment. **(B)** The phosphorylation of ERK determined by western blot in young and aged BMSCs in response to rCCL3 and/or neutralization antibody against Dkk-1 treatment. GAPDH was used as loading control. All the data were obtained from three independent experiments. Data were shown as the means ± s.d. **: $p<0.01$, ***: $p<0.001$, ns: no significance.

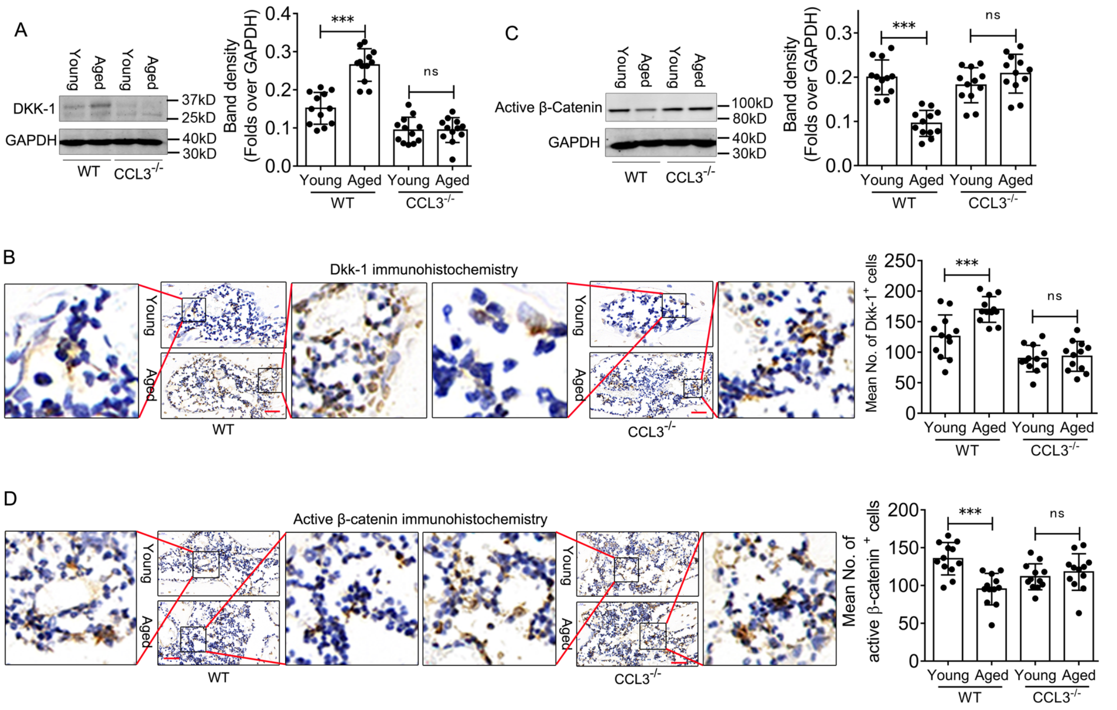

Figure S15

Figure S15: Dkk-1 and active β-catenin expression in the bone marrow and BMSCs from young and aged Ccl3$^{-/-}$ and WT mice. (A) Dkk-1 protein expression determined by western blot in BMSCs from young and aged CCL3$^{-/-}$ and WT mice. **(B)** Dkk-1 expression in femur bone marrow of young and aged CCL3$^{-/-}$ and WT mice determined by immunohistochemistry. **(C)** Active β-catenin protein expression determined by western blot in BMSCs from young and aged CCL3$^{-/-}$ and WT mice. **(D)** Active β-catenin expression in femur bone marrow of young and aged CCL3$^{-/-}$ and WT mice determined by immunohistochemistry. All the data were obtained from three independent experiments. Data were shown as the means ± s.d. **: $p<0.01$, ***: $p<0.001$, ns: no significance.

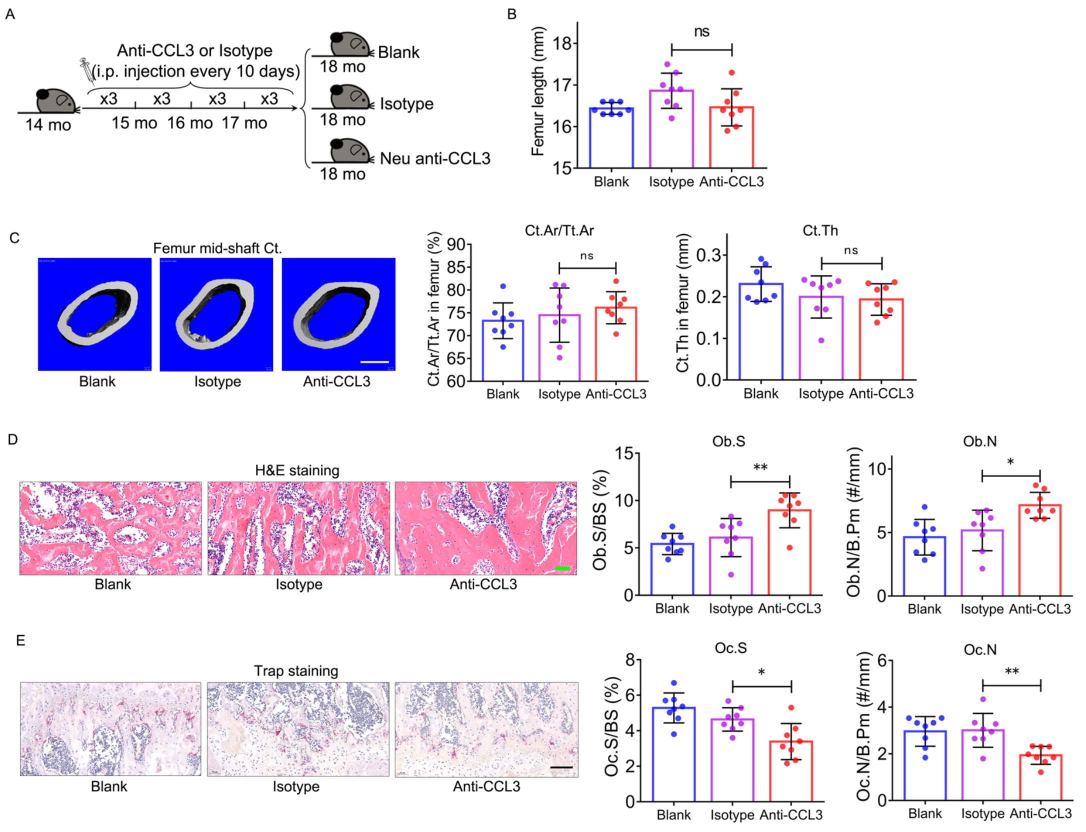

Figure S16

Figure S16: Cortical bone mass and static bone histomorphometry of aged mice in response to neutralization antibody against CCL-3 administration. (A) Neutralization antibody injection procedure. mo: month, Neu: neutralization, i.p.: intraperitoneal. (B) **Femur length of aged mice with neu anti-CCL3 administration (n=8).** (C) Ct.Ar/Tt.Ar and Ct.Th of femur mid-shaft from aged mice with Neu anti-CCL3 injection determined by micro-CT (n=8). (D) H&E staining and the histomorphometric analysis of Ob.S/BS and Ob.N/B.Pm in femur of aged mice with Neu anti-CCL3 injection (n=8). Scale bar: 50 μm. (E) TRAP staining and the quantification of Oc.S/BS and Oc.N/B.Pm of femur of aged mice with Neu anti-CCL3 injection. Scale bar: 100 μm. All the data were obtained from three independent experiments. Data were shown as the means ± s.d. *: $p<0.05$, **: $p<0.01$, ns: no significance.

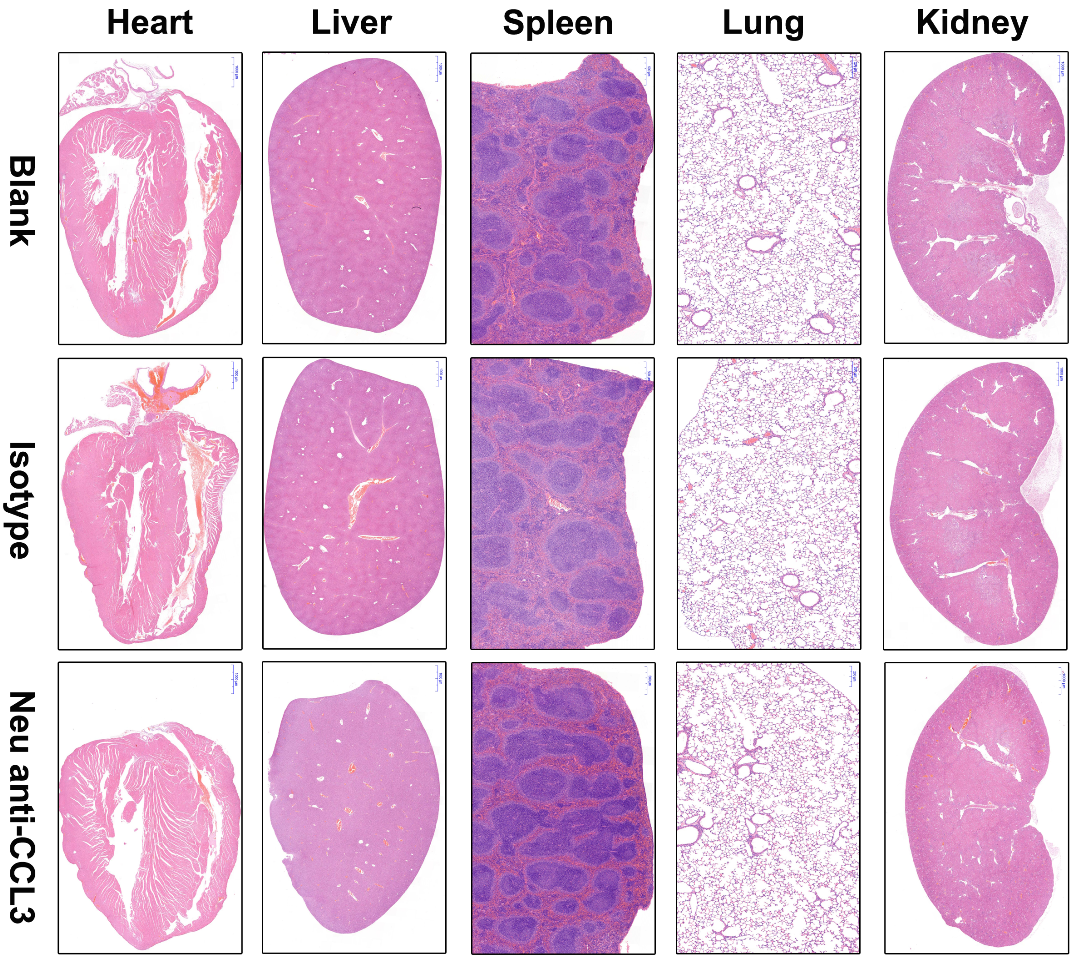

Figure S17

Figure S17: Histological evaluation of core organs of aged mice in response to CCL3 neutralization antibody administration. Core organs including heart, liver, spleen, lung, and kidney were recovered from aged mice in response to CCL3 neutralization antibody or vehicle administration. H&E staining was employed to evaluate the histological phenotype of these organs described above.

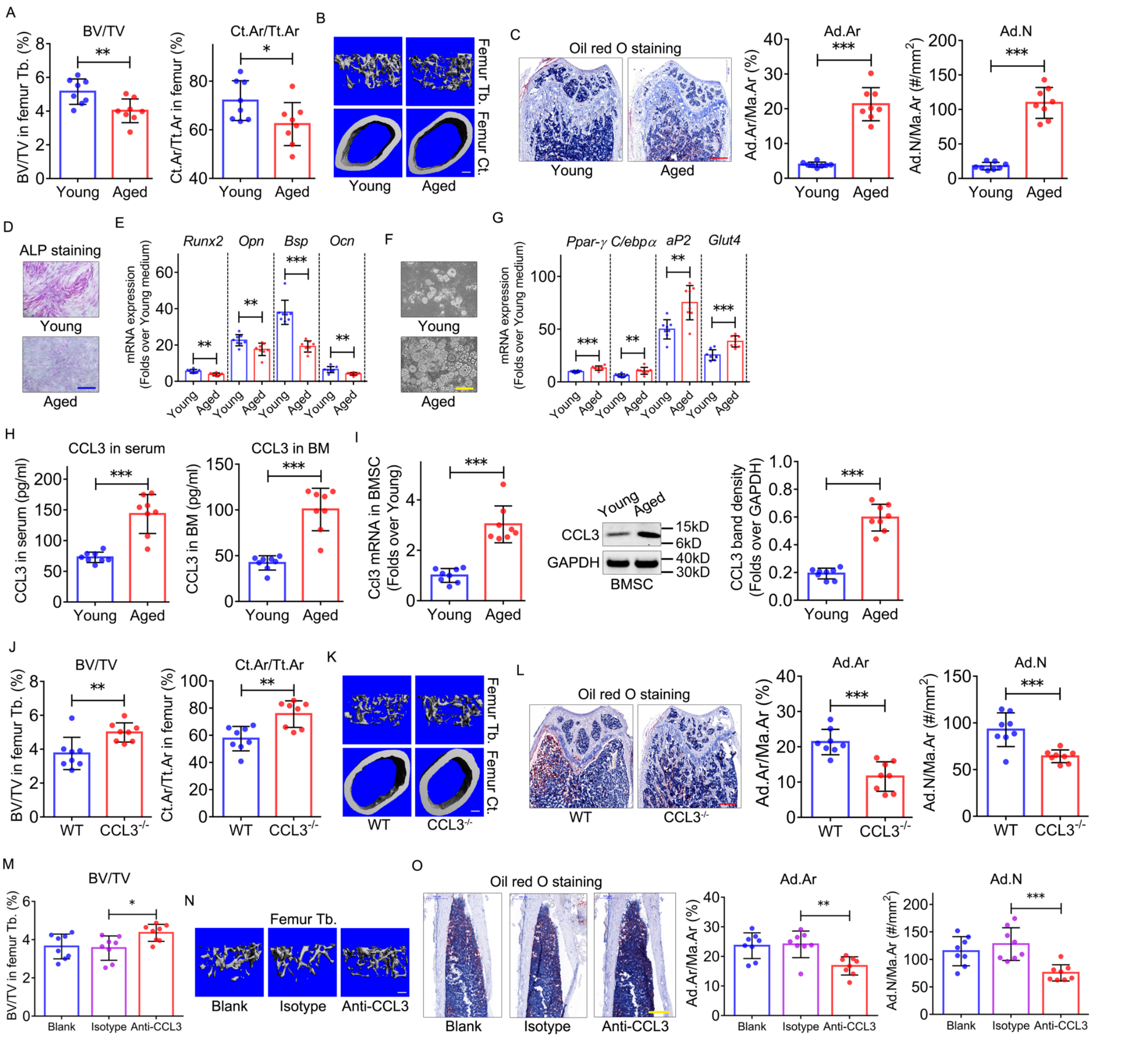

Figure S18

Figure S18: Representative data in female mice. (A) Trabecular bone volume fraction (BV/TV) of distal femur and cortical bone area fraction (Ct.Ar/Tt.Ar) of femur mid-shaft from 6- (young) and 18-month-old (aged) mice determined by micro-CT (n=8). **(B)** Representative micro-CT 3D reconstruction images. Scale bar: 250 μm. **(C)** The quantification of adipocytes area (Ad.Ar/Ma.Ar) and number (Ad.N/Ma.Ar) on the basis of Oil red O staining of femur of young and aged mice (n=8). Scale bar: 500 μm. **(D)** Alkaline phosphatase (ALP) staining of in vitro BMSCs culture from young and aged mice. Scale bar: 20 μm. **(E)** mRNA expression of osteogenesis markers including Runx2, Opn, Bsp and Ocn determined by qRT-PCR when BMSCs from young and aged mice was induced to undergo osteogenic differentiation (n=8). **(F)** Fat droplet formation of in vitro BMSCs culture from young and aged

mice when induced to undergo adipogenic differentiation. Scale bar: 10 μm. **(G)** mRNA expression of adipogenesis markers including PPAR-γ, C/EBPα, aP2 and Glut4 determined by qRT-PCR when BMSCs from young and aged mice was induced to undergo adipogenic differentiation (n=8). **(H)** The levels of CCL3 in peripheral blood serum and femur bone marrow from young and aged mice determined by ELISA (n=8). **(I)** CCL3 mRNA and protein expression in BMSCs from young and aged mice determined by qRT-PCR and western blot (n=8). **(J)** BV/TV of distal femur and Ct.Ar/Tt.Ar of femur mid-shaft from WT and $CCL3^{-/-}$ mice determined by micro-CT (n=8). **(K)** Representative micro-CT 3D reconstruction images. Scale bar: 250 μm. **(L)** The quantification of adipocyte area and number on the basis of Oil red O staining of femur of WT and $CCL3^{-/-}$ mice (n=8). Scale bar: 500 μm. **(M)** BV/TV of distal femur from aged mice with neutralization antibody against CCL3 administration determined by micro-CT (n=8). **(N)** Representative micro-CT 3D reconstruction images. Scale bar: 250 μm. **(O)** The quantification of adipocyte area and number on the basis of Oil red O staining of femur of aged mice with neutralization antibody against CCL3 administration (n=8). Scale bar: 500 μm. All the data were obtained from three independent experiments. Data were shown as the means ± s.d. *: $p<0.05$, **: $p<0.01$, ***: $p<0.001$.

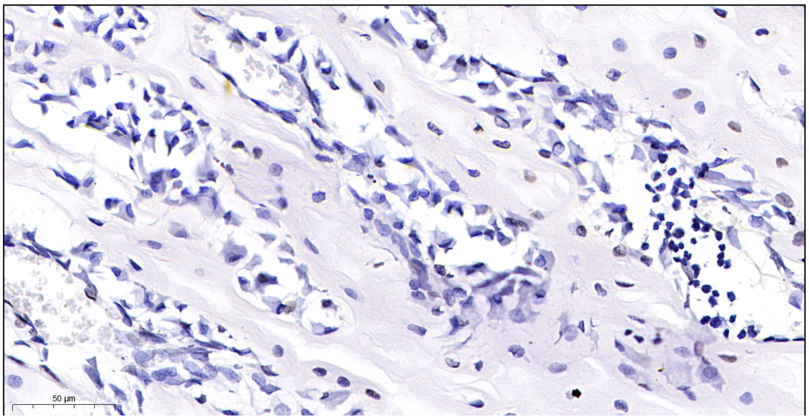

Figure S19

Figure S19: Immunohistochemistry analysis using isotype IgG (negative control) in mice femur. Scale bar: 50 µm.
